# Overcoming selection bias in synthetic lethality prediction

**DOI:** 10.1093/bioinformatics/btac523

**Published:** 2022-07-25

**Authors:** Colm Seale, Yasin Tepeli, Joana P Gonçalves

**Affiliations:** Pattern Recognition & Bioinformatics, Department of Intelligent Systems, Faculty EEMCS, Delft University of Technology, Delft 2628 XE, The Netherlands; Holland Proton Therapy Center (HollandPTC), Delft 2600 AC, The Netherlands; Pattern Recognition & Bioinformatics, Department of Intelligent Systems, Faculty EEMCS, Delft University of Technology, Delft 2628 XE, The Netherlands; Pattern Recognition & Bioinformatics, Department of Intelligent Systems, Faculty EEMCS, Delft University of Technology, Delft 2628 XE, The Netherlands

## Abstract

**Motivation:**

Synthetic lethality (SL) between two genes occurs when simultaneous loss of function leads to cell death. This holds great promise for developing anti-cancer therapeutics that target synthetic lethal pairs of endogenously disrupted genes. Identifying novel SL relationships through exhaustive experimental screens is challenging, due to the vast number of candidate pairs. Computational SL prediction is therefore sought to identify promising SL gene pairs for further experimentation. However, current SL prediction methods lack consideration for generalizability in the presence of selection bias in SL data.

**Results:**

We show that SL data exhibit considerable gene selection bias. Our experiments designed to assess the robustness of SL prediction reveal that models driven by the topology of known SL interactions (e.g. graph, matrix factorization) are especially sensitive to selection bias. We introduce selection bias-resilient synthetic lethality (SBSL) prediction using regularized logistic regression or random forests. Each gene pair is described by 27 molecular features derived from cancer cell line, cancer patient tissue and healthy donor tissue samples. SBSL models are built and tested using approximately 8000 experimentally derived SL pairs across breast, colon, lung and ovarian cancers. Compared to other SL prediction methods, SBSL showed higher predictive performance, better generalizability and robustness to selection bias. Gene dependency, quantifying the essentiality of a gene for cell survival, contributed most to SBSL predictions. Random forests were superior to linear models in the absence of dependency features, highlighting the relevance of mutual exclusivity of somatic mutations, co-expression in healthy tissue and differential expression in tumour samples.

**Availability and implementation:**

https://github.com/joanagoncalveslab/sbsl

**Supplementary information:**

Supplementary data are available at *Bioinformatics* online.

## 1 Introduction

Synthetic lethality (SL) describes a relationship between two genes where simultaneous loss of function in both genes causes cell death, but independent disruption of either gene does not affect cell viability. An SL relationship can be exploited for precision anti-cancer treatment by targeting a gene known to be synthetic lethal with another gene that is deleteriously mutated in the tumour cells. This targeted gene disruption not only induces the death of tumour cells, it is also unlikely to affect healthy cells if they do not carry the mutation. For instance, PARP inhibitor drugs are preferentially lethal towards tumour cells with BRCA1 or BRCA2 mutations and were the first SL-based therapy approved for use in the clinic (Lord *et al.*, 2015). Developing SL-based therapies requires the identification of novel SL interactions through SL loss-of-function screens, which silence gene pairs of interest and measure the respective effect on cell viability (Nijman, 2011). However, exhaustive screening is expensive and becomes impractical due to the vast number of possible gene pairs. This is where computational SL prediction comes into play to guide experimental follow-up and reduce screening to only promising SL pairs.

Previous computational approaches have derived SL through the analysis of gene mutation or expression (Jerby-Arnon *et al.*, 2014; Wan *et al.*, 2020; Wappett *et al.*, 2016), patient survival (Feng *et al.*, 2019; Lee *et al.*, 2018), metabolic networks (Folger *et al.*, 2011; Raman *et al.*, 2018), protein–protein interactions (Jacunski *et al.*, 2015; Kranthi *et al.*, 2013), signalling pathways (Zhang *et al.*, 2015), existing SL networks (Cai *et al.*, 2020; Huang *et al.*, 2019; Liu *et al.*, 2020), evolutionary conservation within and between species (Conde-Pueyo *et al.*, 2009; De Kegel *et al.*, 2021; Lu *et al.*, 2015; Wu *et al.*, 2014), among others. We categorize existing SL prediction approaches as either SL topology-based or SL feature-based methods. Informally, SL topology prediction methods: (i) consider a limited prediction universe based on a predefined set of genes, usually induced by the availability of SL labels; (ii) are explicitly aware of the gene–gene SL label graph structure, where nodes denote genes and edges denote SL relationships between pairs of genes. SL topology methods can be further categorized into matrix factorization techniques like pca-gCMF (Liany *et al.*, 2020), SL2MF (Liu *et al.*, 2020), GRSMF (Huang *et al.*, 2019) and graph-based methods like SLant (Benstead-Hume *et al.*, 2019), DDGCN (Cai *et al.*, 2020) and GCATSL (Long *et al.*, 2021). Conversely, SL feature methods are unaware of gene identity or the structure defined by the SL relationship labels and rely exclusively on molecular features of genes for the prediction task. For this reason, feature models can be used to predict an SL relationship for any pair of genes with a corresponding feature-based representation. SL feature methods include statistical techniques like DAISY (Jerby-Arnon *et al.*, 2014) and BiSEp (Wappett *et al.*, 2016), and supervised learning models such as Lu *et al.* (2015), DiscoverSL (Das *et al.*, 2019) and EXP2SL (Wan *et al.*, 2020).

Significant challenges remain before existing SL prediction methods can be routinely used to guide experimental screening. To be effective, they must rank positive SL gene pairs consistently high across multiple datasets, be able to make predictions for unseen genes, and generalize to unseen gene pairs. However, most studies assess prediction performance under limited scenarios, for instance focusing on a single cancer type and testing of gene pairs whose genes individually appear in the training set. We hypothesize that some genes may be overrepresented in existing SL labels while others remain understudied for historical or academic reasons (Stoeger *et al.*, 2018). The extreme case, where SL labels are available for many pairs but involving only a few genes, is also likely to induce SL relationship biases because pairs involving the same gene are not independent from each other. We argue that the presence of strong biases in SL labels can lead to performance overestimation, particularly of SL topology models which are explicitly designed to exploit them.

In this work, we propose different experiments to assess the sensitivity of SL prediction methods to selection biases. We also introduce SBSL (selection bias-resilient synthetic lethality) prediction models, with two main goals in mind: (i) improving model resilience to biases in SL prediction; and (ii) bridging the performance gap between SL topology and SL feature methods currently perceived in the SL prediction literature. To improve bias-resilience, we propose SL feature models based on supervised machine learning (ML) that explicitly ignore the structure of the SL label graph. To improve performance, we define novel features based on molecular data that could be relevant for SL prediction but remains underexplored in the SL prediction context. Specifically, these are the interaction between gene dependency scores (measuring cell viability upon gene silencing) and mutations in cancer cell lines (Behan *et al.*, 2019; Dempster *et al.*, 2019; McFarland *et al.*, 2018; Meyers *et al.*, 2017), as increased dependency on one gene in cell lines harbouring a deleterious mutation in another gene may indicate SL between the two; gene expression from healthy donor tissue, in addition to expression from patient tumour tissue, which could help identify tumour-specific changes in the relationship between the pair of genes; measures of mutual exclusivity, quantifying the non-co-occurrence of mutations in a pair of genes (Babur *et al.*, 2015; Canisius *et al.*, 2016); change in survival time between cancer patients with and without mutations or aberrant expression in the pair of genes, both of which may be associated with SL (Lee *et al.*, 2018; Srihari *et al.*, 2015).

## 2 Materials and methods

Our proposed models aim to predict if a given pair of genes is synthetically lethal for a specific cancer type, where the pair is described by a collection of molecular features. We approach it as a binary classification problem.

### 2.1 Data

*SL labels.* We obtained cancer-specific SL labels from two studies, ISLE and DiscoverSL (Das *et al.*, 2019; Lee *et al.*, 2018). Together, they included thousands of SL relationships experimentally derived by 21 other studies using double gene knockdown/knockout experiments or targeting of one gene using CRISPR or RNAi in contexts where the other gene is either endogenously inactive or rendered inactive through the use of drug compounds. We removed duplicate gene pair entries from ISLE and DiscoverSL separately by retaining a single entry if all entries agreed, or removing all duplicate entries if any of them disagreed on the label. To combine the two datasets, we reduced 63 gene pairs with duplicate entries across the datasets to a single entry per pair. In case of disagreement, we chose the label from DiscoverSL, since there was a lower level of disagreement within DiscoverSL than within ISLE. We ended up with 7962 labelled gene pairs distributed over the four cancer types that had at least 200 positive and negative labels after pre-processing, namely breast (BRCA), colon (COAD), lung (LUAD) and ovarian (OV; Table 1). The ISLE, DiscoverSL and combined SL gold standards had differing cancer type representations and class imbalances. We used the combined SL gold standard in our experiments except where otherwise specified.

**Table 1.** SL gold standard statistics. Breakdown of labels into positives and negatives, unique gene count and percentage of labelled pairs

	ISLE		DiscoverSL		Combined			
	+	−	+	−	+	−	No. of genes	Labelled (%)
BRCA	713	1168	835	72	1548	1240	1072	0.39
COAD	859	806	0	0	859	806	1560	0.14
LUAD	202	5155	347	312	549	5467	804	1.66
OV	223	449	0	0	223	449	86	18.14
All	1997	7578	1182	384	3179	7962	3072	0.05

Columns + and − show number of positive and negative labels for each dataset. Number of genes and Labelled (%) denote the number of unique genes and percentage of labelled pairs (of all possible pairs involving genes from the combined dataset).

*Cancer cell line data.* We used cancer cell line gene dependency scores based on CRISPR (CERES; Dempster *et al.*, 2019; Meyers *et al.*, 2017) and RNA interference (DEMETER2; Behan *et al.*, 2019; McFarland *et al.*, 2018) screens from the 19Q3 DepMap and DEMETER2 Data v6 public releases, respectively. We also obtained functionally categorized mutation data per gene (Ghandi *et al.*, 2019).

*Patient tumour and clinical data.* We collected the following patient tumour sample data from The Cancer Genome Atlas (TCGA) using the Broad GDAC Firehose pipeline run *stddata__2016_01_28* (TCGA GDAC, 2016): mutation data, discrete copy-number variation (CNV) scores from GISTIC (Mermel *et al.*, 2011), patient race, age, sex and survival time (days). We also obtained gene expression data from the GEO (accession GSM1536837) as aggregated read counts (Rahman *et al.*, 2015).

*Healthy tissue data.* We collected expression data from GTEx for breast, lung, colon and ovarian tissue of healthy donors, provided as gene-aggregated transcripts per million (TPM) values (dbGaP accession phs000424.v8.p2; Lonsdale *et al.*, 2013). We also included expression data of TCGA matched normal BRCA and LUAD samples from GEO, as described for patient tumours.

*Biological pathway data.* We downloaded KEGG (Kanehisa and Goto, 2000), PID (Schaefer *et al.*, 2009) and Reactome (Jassal *et al.*, 2020) pathway gene sets from the Molecular Signatures Database v7.1 (MSigDB; Liberzon *et al.*, 2011; Subramanian *et al.*, 2005).

*Protein–protein interaction and gene ontology data.* Protein–protein interaction data were downloaded from STRINGdb, version 11 (Szklarczyk *et al.*, 2021). We selected only interactions supported by curated experimental evidence. Gene Ontology (GO) biological process and cellular component data were downloaded from the GO repository on March 18, 2021 (Ashburner *et al.*, 2000; The Gene Ontology Consortium, 2021).

### 2.2 Features

Every example denotes a tissue type-specific relationship between a pair of genes (A, B), characterized by the following 27 molecular features (see Supplementary Table S1 for a summary of all individual features).

*Gene dependencies.* We calculated five features for each type of gene dependency, CRISPR or RNAi (10 in total). We performed two two-tailed Wilcoxon rank-sum tests (Mann and Whitney, 1947), one for (A, B) and another for the same pair in reverse order (B, A). Each test quantifies the change in dependency on the first gene between cell lines with and without a non-silent mutation in the second gene. We chose as features the test statistic and *P*-value for the tested pair (A, B) or (B, A) that yielded the smallest *P*-value. We defined two additional features as the Pearson’s correlation coefficient and corresponding two-tailed *t*-test *P*-value between the dependency scores of A and B. The fifth feature was the average of the means of the dependency scores for genes A and B. Respectively, the features are termed *CRISPR/RNAi_dep_stat*, *CRISPR/RNAi_dep_pvalue*, *CRISPR/RNAi_cor_stat*, *CRISPR/RNAi_cor_pvalue* and *CRISPR/RNAi_avg*.

*Mutual exclusivity.* We calculated seven mutual exclusivity features based on tumour mutation data using three methods: DiscoverSL (four features; Das *et al.*, 2019), DISCOVER (Canisius *et al.*, 2016) and MUTEX (Babur *et al.*, 2015). These features are termed *discoversl_mutex_amp*, *discoversl_mutex_del*, *discoversl_mutex_mut*, *discoversl_mutex*, *discover_mutex* and *MUTEX*. We calculated an additional mutual exclusivity *P*-value, *mutex_alt*, by treating every non-silent mutation, amplification (CNV = 2), and deletion (CNV = -2) as an ‘alteration’ event. We used a hypergeometric test:

(1) $$p=1-\sum_{j=n_{A,B}}^{\min(n_{A},n_{B})}\frac{\left(\begin{array}{c} n_{A}\\ j \end{array}\right)\left(\begin{array}{c} n_{T}-n_{A}\\ n_{B}-j \end{array}\right)}{\left(\begin{array}{c} n_{T}\\ n_{B} \end{array}\right)},$$

where $n_{A}$ and $n_{B}$ are the numbers of tumour samples with an alteration in A and B, respectively, $n_{A,B}$ is the number of samples with alterations in both, and $n_{T}$ is the total number of samples.

*Survival.* We modelled patient survival time using Cox proportional hazard models accounting for the alteration status of gene pair (A, B) in patient tumours. We defined the status as ‘altered’ if any of the following alterations occur in both A and B (unaltered otherwise): copy-number amplifications (CNV = 2) or deletions (CNV = −2), non-silent mutations or aberrant expression. We defined aberrant expression as having a gene expression level in the upper or lower fifth percentile across all patient samples. We also controlled for age, race and sex as follows:

(2) $$\begin{array}{c} ln\;h(t)∼ln\;h_{0}+\beta_{1}s(A,B)+\beta_{2}\text{sex}+\beta_{3}\text{age}+\beta_{4}\text{race} \end{array},$$

where h(t) is the hazard function defined as the conditional probability of a patient dying at time *t* given that the patient has survived to time *t* (Bewick *et al.*, 2004). The indicator variable s(A,B) denotes the alteration status of gene pair (A, B) in a patient tumour sample. The β values are the regression coefficients. One feature, *logrank_pval*, was defined as the two-tailed *P*-value of $\beta_{1}\neq 0$ using the Wald statistic (Bangdiwala, 1989).

*Co-expression.* We determined co-expression between a gene pair for three types of biological samples: tumour and normal TCGA samples (for BRCA and LUAD), and healthy donor GTEx samples. We used pairwise Pearson’s correlations and two-tailed *t*-test *P*-values, yielding four to six features: *tumour_corr/pvalue*, *normal_corr/pvalue* and *gtex_corr/pvalue*.

*Differential expression.* We calculated differential expression using tumour samples to quantify the variation in expression of one gene given the presence or absence of non-silent mutations in the other gene. We performed two differential expression tests per gene pair (A, B), for gene A based on the mutation status of gene B and vice versa, and used the minimum of the two *P*-values and the corresponding log fold-change as features for the gene pair. These were calculated using edgeR based on the read count data (Robinson *et al.*, 2010). Using the edgeR default parameter values, we performed Trimmed Mean of *M*-values normalization and calculated gene-wise log2 fold-changes and *P*-values as features, respectively, termed *diff_exp_logFC* and *diff_exp_pvalue*.

*Pathway co-participation.* We calculated a *pathway_coparticipation P*-value denoting the significance of co-occurrence of a pair of genes in a set of pathways using a hypergeometric test as defined in Equation (1). Here, $n_{A}$ and $n_{B}$ are the number of occurrences of genes A and B in all pathways, respectively, $n_{A,B}$ is the number of occurrences of both genes in the same pathway and $n_{T}$ is the total number of pathways. The set of pathways was defined as the union of the KEGG, PID and Reactome gene sets.

### 2.3 SL prediction models

*SBSL prediction models.* We trained logistic regression and random forest models with regularization, as representatives of linear and non-linear models. For logistic regression, we used L0 and L2 (L0L2), or L1 and L2 (Elastic Net) regularization, as implemented, respectively, in the L0Learn and glmnet packages (Friedman *et al.*, 2010; Hazimeh and Mazumder, 2020). We also tried two regularized random forest implementations: Multivariate random forests with Unbiased Variable selection in R (MUVR; Shi *et al.*, 2019), and Regularized Random Forests (RRF; Deng and Runger, 2012). MUVR combines a random forest model with feature selection through repeated, nested, cross-validation and backward feature elimination on the train set. RRF is a random forest variant that uses two parameters to control model complexity: *mtry* determining how many features are randomly sampled at each new node; and *coefReg* to control the penalization of the information gained when adding a new feature to the model to split at a given node.

*Other SL prediction models for comparison.* We compared the SBSL models against five other published methods: statistical approach DAISY (Jerby-Arnon *et al.*, 2014), supervised model DiscoverSL (Das *et al.*, 2019), graph-based GCATSL (Long *et al.*, 2021), GRSMF (Huang *et al.*, 2019) and matrix factorization pca-gCMF (Liany *et al.*, 2020).

### 2.4 Training and evaluation

For each experiment, we created 10 different train/test set splits of the available dataset(s), so that we could better assess the robustness of the models. For each pair of train and test sets, which we term run for short, we performed the following steps: hyperparameter search on the train set using cross-validation, learning of a final model on the entire train set using the best parameters, and assessing the final model on the corresponding disjoint test set. We report the averages and standard deviations of our performance evaluation metrics across the 10 runs. These steps are further detailed below.

*Train and test sets.* All pairs of train and test sets were created as follows, unless otherwise specified. To handle class imbalances (Table 1), we uniformly downsampled the dataset to ensure an equal number of SL and non-SL pairs. We then divided it into train and test sets with a 70/30 split via uniform sampling. We standardized every feature in both sets by subtracting the mean and dividing by the standard deviation calculated from the train set. We also excluded any feature for which at least 95% of the values in its feature vector were constant.

*Hyperparameter tuning.* To select model hyperparameters for Elastic Net and RRF, we defined a search space per model as follows; Elastic Net: *lambda* = [0, 1], *alpha* = [0, 1]; RRF: *mtry* = [4, 8], *coefReg* = [0.5, 1]. For L0L2, the search space hyperparameters were set to *nGamma *=* *20 and *nLambda *=* *50. For the Elastic Net, RRF and L0L2 models, we conducted 10-fold cross-validation on the train set with five repeats using the area under the receiver operating characteristic (AUROC) as performance metric. Results of the hyperparameter search for these three models can be found in Supplementary Figures S1–S3. The hyperparameters used for the MUVR backwards feature elimination algorithm were *nRep *=* *5, *nOuter *=* *10, and *varRatio *=* *0.8.

*Evaluation.* Following hyperparameter tuning, SBSL models were trained on the entire train set using the best hyperparameters. Performance was then assessed on the corresponding disjoint test set, using receiver operating characteristic (ROC) and precision-recall (PR) curves. The curves were summarized by AUROC or area under the precision-recall curve (AUPRC) metrics. We report averages and standard deviations of the AUROC and AUPRC across the 10 runs.

*Comparison with other SL prediction methods.* We calculated DAISY scores for all gene pairs, and predicted DiscoverSL scores for test set pairs using the package provided by the authors. GCATSL, GRSMF and pca-gCMF models were trained on the train set using their default parameter settings (see Supplementary Methods). Scores obtained by all methods for gene pairs in the test set were used for comparison during evaluation.

*Feature importance.* We calculated permutation feature importance (FI) values for SBSL models based on the test set to determine which features contributed most to the predictions (Fisher *et al.*, 2019). Interpreting FI scores can be confounded by multicollinearity, as importance may spread over correlated features. For this reason, we assessed multicollinearity using variance inflation factors (VIF; James *et al.*, 2013).

## 3 Results and discussion

### 3.1 SBSL and SL topology methods are the top performers

We first evaluated the performance of the SL prediction models separately within each cancer type (BRCA, COAD, LUAD and OV). We evaluated the predictive performance of the SBSL logistic regression (L0L2, Elastic Net) and random forest (MUVR, RRF) models against published methods DAISY, DiscoverSL, GCATSL, GRSMF and pca-gCMF.

On BRCA and LUAD, the SBSL models and the matrix factorization methods GRSMF and pca-gCMF performed most consistently considering the two metrics, with average AUROC and AUPRC above 0.80 (Tables 2 and 3, Supplementary Fig. S4 for ROC and PR curves). SBSL models did better at predicting true SL pairs for BRCA and LUAD than the other approaches, with the exception of pca-gCMF on BRCA (Table 3). GRSMF performed reasonably with average AUPRC above 0.80, but GCATSL performed poorly on BRCA (average AUPRC of 0.55) while scoring highest among the SL Topology methods on LUAD (average AUPRC of 0.85). On COAD, AUROC performances were very modest across the board, with SBSL models featuring on the higher end (0.38 < average AUROC < 0.64).

**Table 2.** Classification performance of SL prediction models within a cancer type, denoted by the AUROC curve

Method	BRCA	COAD	LUAD	OV
Elastic Net	0.84 ± 0.01	0.60 ± 0.02	0.85 ± 0.02	0.59 ± 0.03
L0L2	0.84 ± 0.01	0.60 ± 0.02	0.85 ± 0.02	0.59 ± 0.03
MUVR	0.86 ± 0.01	**0.64** **± 0.01**	**0.87** **±** **0.01**	0.56 ± 0.07
RRF	0.86 ± 0.01	0.63 ± 0.02	0.87 ± 0.02	0.57 ± 0.07
DAISY	0.61 ± 0.02	0.38 ± 0.02	0.44 ± 0.03	0.41 ± 0.04
DiscoverSL	0.54 ± 0.02	0.54 ± 0.02	0.54 ± 0.03	0.45 ± 0.04
GCATSL	0.59 ± 0.04	0.51 ± 0.01	0.86 ± 0.03	**0.99** **±** **0.02**
GRSMF	0.82 ± 0.01	0.57 ± 0.02	0.87 ± 0.02	**0.99** **±** **0.01**
pca-gCMF	**0.90** **±** **0.02**	0.54 ± 0.03	0.87 ± 0.02	0.94 ± 0.05

Mean and standard deviations of AUROC performance values over 10 runs. Bold text indicates the method (row) with the largest mean AUROC value for each cancer type (column).

**Table 3.** Classification performance of SL prediction models within a cancer type, denoted by the AUPRC

Method	BRCA	COAD	LUAD	OV
Elastic Net	0.87 ± 0.01	0.59 ± 0.01	**0.87** **±** **0.02**	0.58 ± 0.03
L0L2	0.88 ± 0.01	0.59 ± 0.02	**0.87** **±** **0.02**	0.58 ± 0.04
MUVR	**0.89** **±** **0.01**	0.62 ± 0.02	**0.87** **±** **0.02**	0.54 ± 0.06
RRF	**0.89** **±** **0.01**	**0.63** **±** **0.02**	**0.87** **±** **0.02**	0.55 ± 0.05
DAISY	0.58 ± 0.02	0.43 ± 0.01	0.47 ± 0.02	0.48 ± 0.04
DiscoverSL	0.55 ± 0.02	0.53 ± 0.02	0.55 ± 0.03	0.49 ± 0.04
GCATSL	0.55 ± 0.02	0.50 ± 0.01	0.82 ± 0.04	**0.98** **±** **0.03**
GRSMF	0.81 ± 0.01	0.59 ± 0.02	0.85 ± 0.02	0.97 ± 0.04
pca-gCMF	**0.89** **±** **0.04**	0.56 ± 0.03	0.83 ± 0.03	0.93 ± 0.06

Mean and standard deviations of AUPRC performance values over 10 runs. Bold text indicates the methods (rows) with the largest mean AUPRC value for each cancer type (column).

On OV, the SBSL models predicted poorly whereas GCATSL, GRSMF and pca-gCMF showed high AUROC and AUPRC scores above 0.90. We hypothesize that the low performance of SBSL models in OV could be due to the modest mutational burden typically observed for this cancer type (Vareki, 2018), which could affect the resolution and informativeness of features relying on mutation data. We confirmed that OV cell lines contained a much lower average number of mutations per gene pair than the other cancer types (OV: 1.6, BRCA: 4.33, LUAD: 11.35, COAD: 5.97). As for the high performance of SL topology methods on OV, we reasoned that it could be due to selection bias, which we investigate in a later section.

DAISY and DiscoverSL performed poorly overall and were excluded from subsequent experiments. We note that DAISY is not an ML approach and does not involve separate training and prediction. For fairness, DAISY was applied to the entire dataset, per cancer type, and then evaluated on the same test sets as the other models (see Section 2).

Our results suggest that SBSL models and pca-gCMF are the most consistent and thus may be better suited for pre-selecting SL pairs for experimental follow-up in BRCA and LUAD. Most methods struggled to predict SL for COAD according to one or both performance metrics.

We advance that low mutational burden could negatively affect the performance of SBSL models on OV, and go on to further investigate a possible link between selection bias and the high performance of SL topology methods.

### 3.2 Selection bias drives SL topology method predictions

Since SL topology methods are driven by the structure of the SL label graph, we hypothesized that their predictive performance could be affected by selection bias in SL screens. We sought to assess the impact of this bias.

**Fig. 1.**
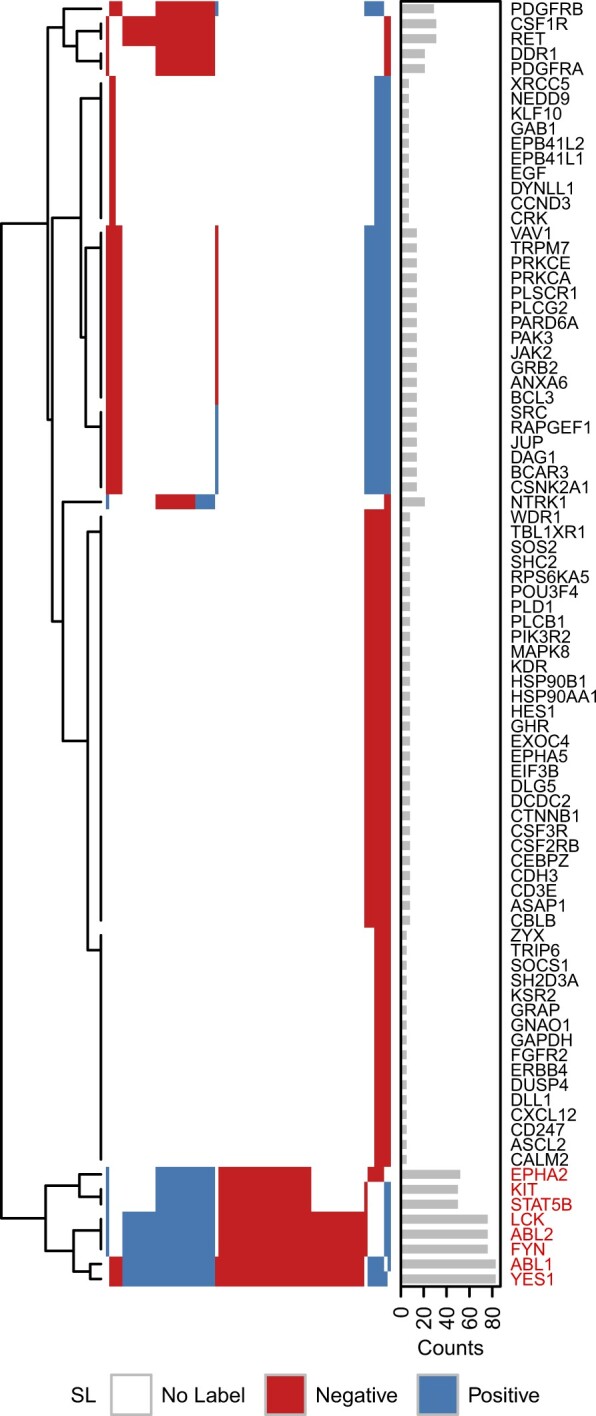
Structure of SL labels. Adjacency plot showing OV gene pairs. Elements along horizontal and vertical axes represent unique genes. Each coloured cell denotes a negative (red) or positive (blue) SL pair. White cells denote pairs with no label. Rows are ordered according to hierarchical clustering with complete linkage and Euclidean distance. Columns follow the ordering of rows. The barplot to the right shows the number of pairs each gene is involved in. The group of eight genes at the bottom of the plot (highlighted in red) consists mostly of tyrosine kinases (A color version of this figure appears in the online version of this article.)

*Selection bias in SL labels.* We examined the coverage and structure of SL labelled gene pairs. The OV set of labelled pairs stood out from the other cancer types for three reasons. First, it had limited gene coverage, comprising only 86 unique genes whereas the other cancer types included 804 to 1560 labelled genes. Second, 18.14% of all possible pairs formed by these 86 genes were labelled in OV, compared to a maximum of 1.66% for the other cancer types (Table 1). Third, the structure of the labels was quite striking: many rows were nearly identical to one another, showing very consistent patterns of SL and non-SL relationships with the same genes. These formed visibly distinct groups, indicative of heavy gene selection bias (Fig. 1). For example, the eight genes at the bottom of Figure 1 (highlighed in red) are functionally related: they mostly consist of tyrosine kinases, which are all reported targets of the same drug dasatinib (Korashy *et al.*, 2014).

The high performances of matrix factorization and graph-based methods on OV data could be expected, given that they are designed to exploit this structure. However, the consistency of patterns seen in these OV labels will not likely generalize well to most randomly selected pairs of genes. The SL labels for the other cancer types exhibited similar bias, albeit less pronounced given the larger sample size and gene coverage (Table 1, Supplementary Figs S5–S8). As an example, for BRCA the five most frequently occurring genes were involved in 52% of all SL labelled gene pairs (PARP1 18%, BRCA1 12%, PTEN 11%, TP53 7% and BRCA2 4%). We also identified two distinct groups of genes with visibly coordinated patterns, which also happened to be functionally related: one group comprised members of cell proliferation pathways (JAK2, GATA3, PIK3C3, FLI1, MAP2K4, PPARA, BIRC3, CREBBP, KRAS, MAP3K1 and others), and the other group contained genes involved in DNA damage response (CHD1, USP6, CANT1, ERCC4, MAML2, DHRS13 and FHIT).

**Fig. 2.**
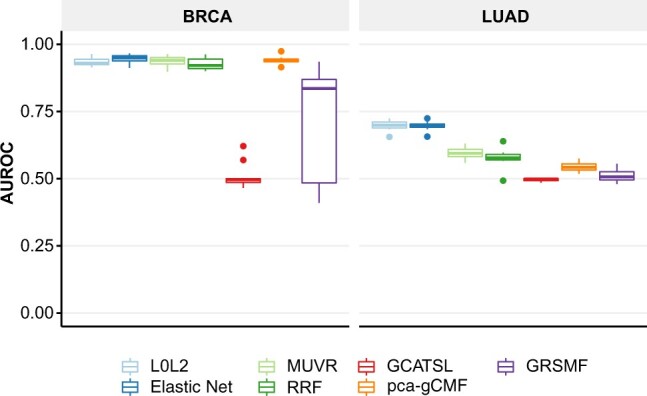
Cross-SL gold standard performances. AUROC values averaged over 10 runs for: (left) BRCA models trained on ISLE and tested on DiscoverSL; (right) LUAD models were trained on DiscoverSL and tested on ISLE

*Cross-SL-dataset generalization.* We assessed the impact of selection bias on the ability of SL prediction methods to generalize across the two datasets of SL labels. We trained BRCA models on gene pairs from ISLE and tested them against DiscoverSL. We also trained LUAD models on DiscoverSL and tested them against ISLE. We focused on these specific combinations, since the number of available SL pairs was insufficient for the reverse combinations. Any labelled pairs present in both datasets were removed from the train set. Our results showed that SBSL models generalized better across gold standards (Fig. 2, Supplementary Fig. S9), with linear models performing best overall. The SL topology approaches (GCATSL, GRSMF and pca-gCMF) struggled to generalize, and their performances decreased to nearly random on LUAD data. We found that pca-gCMF did only marginally better than the graph-based methods on LUAD, but was comparable to our SBSL models on BRCA.

Contributing to the poor performance of SL topology models is the fact that these techniques have difficulty making connections to genes that are not involved in pairs in the train set, an issue that is most prevalent in LUAD SL data. Specifically, for BRCA, 522 of the 907 pairs in DiscoverSL contained genes that also appeared in ISLE. However, for LUAD, only 19 out of 659 DiscoverSL pairs shared a gene with ISLE (Supplementary Figs S10 and S11). SL topology methods would be more affected by this than SBSL models due to missing prior SL information for the genes in the test set.

*Gene holdout experiments.* We further investigated the impact of selection bias on SL prediction using *gene holdout* experiments, where train/test sets were constructed in three different ways, seeking to control the number of genes shared between the two sets. In our original baseline scenario, also termed *None*, we only ensured that there was no overlap in gene pairs between train and test sets. For *Single* holdout, we constructed the train and test sets such that for every gene pair in the test set, only one of the genes from the pair could be present in the train set. For *Double* holdout, we created the train and test sets such that they did not share any genes. Note that there was not enough OV data to conduct the *Double* experiment.

**Fig. 3.**
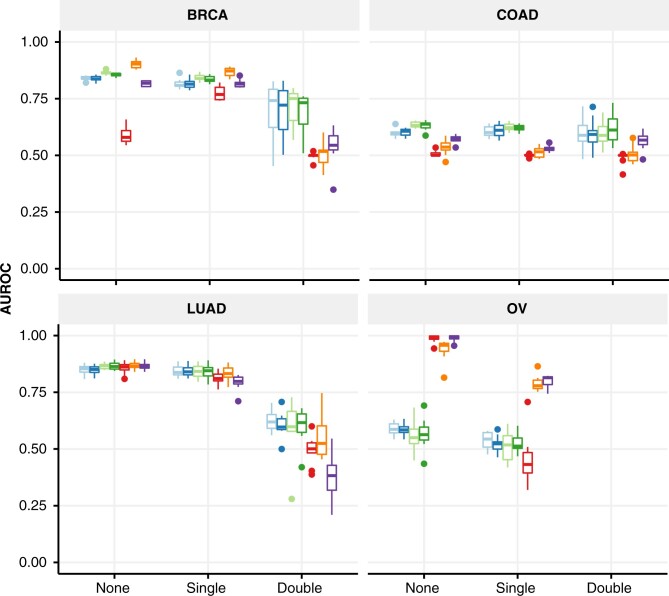
Performances of gene holdout experiments, where bias is controlled by ensuring that none, one or both genes of pairs in the test set are excluded from the train set. Shown are AUROC values for each gene-holdout experiment per cancer type (10 runs). For ‘None’, we only guarantee that train and test sets are disjoint in terms of gene pairs, not individual genes; for ‘Single’, only one gene from a gene pair in the test set can be present in the train set; for ‘Double’ neither gene of a pair in the test set appears in the train set. The results for ‘None’ correspond to those also reported in Table 2. *Note*: There was insufficient data to conduct the OV ‘Double’ experiment

The SBSL models were more robust to gene holdouts than SL topology models on BRCA, COAD and LUAD. For these cancers, SBSL models showed a negligible decrease between baseline and *Single* holdout, and a more pronounced drop to mean AUROC values between 0.60 and 0.75 using *Double* holdout. Comparatively, the performance of SL topology models varied more and became approximately random with *Double* holdout (Fig. 3, Supplementary Fig. S12). OV was the exception, where SL topology methods seemed to do better, possibly due to the previously described bias in SL labels. We note that our results are confounded by shrinking of the train set size as we move through the scenarios from *None* to *Double* and that OV is the smallest of the datasets.

### 3.3 Not all cancers are equal in SL prediction

We wondered whether the underlying molecular patterns that allow us to recognize when two genes are synthetically lethal could be independent of cancer type and thus generalizable across cancers. To answer this question, we assessed the potential benefits of training pan-cancer L0L2 and MUVR models, which could also help mitigate the sparsity and selection bias affecting some of the cancer types (Supplementary Tables S2 and S3 and Figs S13–S14 for results including all SBSL models).

First, we trained two pan-cancer models on data from all four cancer types (Table 4). One was an unbalanced model, with gene pairs uniformly selected from the combined dataset to keep the original cancer type ratios. The other model was trained with balanced proportions of cancer types and class labels by undersampling. Both models were evaluated against held-out data from every cancer type. Model performances improved in almost every case when training on balanced compared to unbalanced data. However, training on multiple cancers resulted in a degradation of overall performance relative to the cancer-specific models. We note that balanced models typically had less gene pairs to train on.

**Table 4.** Performance of one-cancer and pan-cancer models (AUROC)

Method	Cancer	Pan-cancer		One-cancer
		Unbalanced	Balanced	
L0L2	BRCA	0.64 ± 0.02	0.75 ± 0.01	**0.83** **±** **0.01**
	COAD	0.52 ± 0.02	0.51 ± 0.02	**0.60** **±** **0.02**
	LUAD	0.73 ± 0.03	0.79 ± 0.02	**0.83** **±** **0.02**
	OV	0.40 ± 0.04	0.53 ± 0.04	**0.58** **±** **0.03**
MUVR	BRCA	0.76 ± 0.01	0.82 ± 0.02	**0.86** **±** **0.01**
	COAD	0.62 ± 0.02	0.60 ± 0.01	**0.64** **±** **0.01**
	LUAD	0.81 ± 0.02	0.83 ± 0.02	**0.86** **±** **0.01**
	OV	**0.55** **±** **0.06**	0.52 ± 0.04	0.54 ± 0.07

Mean and standard deviation of AUROC values calculated over 10 runs. One-cancer and pan-cancer models trained with unbalanced or balanced cancer representation, tested on held-out gene pairs. Bold text indicates the setting leading to the largest mean AUROC (pan-cancer unbalanced, pan-cancer balanced, or one-cancer).

**Fig. 4.**
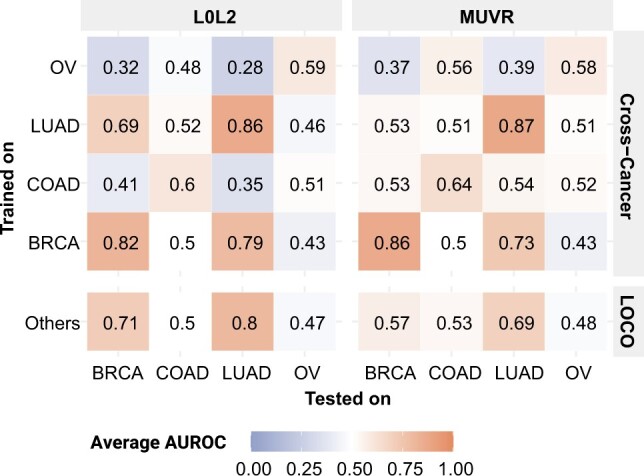
Cross-cancer and LOCO performances. Average AUROC for L0L2 and MUVR models over 10 runs. *Cross-cancer:* Vertical and horizontal axes denote the cancer types used to train and test, respectively. *LOCO:* Horizontal axis denotes the cancer type held out for testing. Models trained on balanced data from all other cancers

We then assessed the ability of SBSL cancer-specific models to make SL predictions for other cancer types (Fig. 4). As expected, models that performed poorly within the same cancer type, like COAD and OV, could not generalize to other cancer types either. The better-performing models, BRCA and LUAD, could not predict well on COAD and OV either. Notably, the L0L2 linear models generalized reasonably both when trained on BRCA and tested against LUAD (mean AUROC 0.79) and vice versa (0.69). The MUVR random forests could generalize when trained on BRCA and tested against LUAD (0.73), but not vice versa (0.53), showing they were more prone to overfit.

We also investigated the ability of models trained on multiple cancers to make SL predictions for unseen cancer types. In this leave-one-cancer-out (LOCO) experiment, we held out one cancer type for testing and trained models using samples from the other three, with balanced class labels and cancer types (Fig. 4, bottom row). The results were consistent with those of the cross-cancer experiment (Fig. 4). For example, the three-cancer models trained on COAD, LUAD and OV generalized to BRCA as well as the LUAD-specific models. This indicates that training on multiple cancer types is not necessarily detrimental to cross-cancer generalization.

### 3.4 Gene dependency-based features are most important

We used permutation feature importance scores (PFI) to quantify the contribution of the 27 features to the predictions of our SBSL models. To obtain meaningful PFI scores, the models should be reasonably accurate, thus we excluded the lower-performing OV and COAD models (Table 2).

Gene dependency-based features were the largest contributors to the performance of BRCA and LUAD models (Supplementary Figs S15 and S16). The highest ranked feature overall was *CRISPR_dep_stat*, which quantifies the change in dependency score of one gene between cell lines with and without a non-silent mutation in the other gene. Specifically, for linear models, the importance of *CRISPR_dep_stat* was nearly 2-fold greater than the importance of the second-ranked feature. Ranking second were features denoting the average of means of gene dependency scores across all cell lines. For all LUAD models and the BRCA random forest models, the choice went to the CRISPR-based feature (*CRISPR_avg*), while BRCA logistic regression models picked the RNAi-based feature (*RNAi_avg*). Even though CRISPR and RNAi-based dependency scores exhibit some differences, they are still moderately to highly correlated (multicollinearity VIF >2, Supplementary Table S4), and thus fairly equivalent in contribution to SL prediction.

**Fig. 5.**
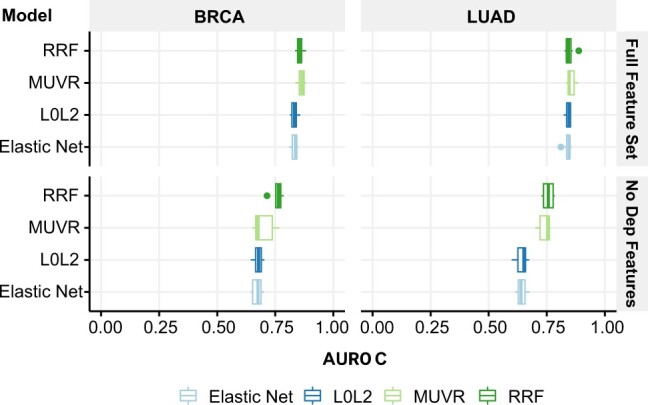
Performance of SBSL models with and without gene dependency-based features (AUROC over 10 runs), respectively, labelled ‘Full Feature Set’ and ‘No Dep Features’

To further assess the reliance of our SBSL models on dependency-based features, we retrained and tested BRCA and LUAD models without these features. This led to a significant decrease in mean AUROC across all models, from between 0.83 and 0.85 to between 0.64 and 0.76, for both cancer types (Fig. 5). We also calculated PFI values for these models, which showed not only higher variability but also a few clear patterns. The DISCOVER mutual exclusivity score (Canisius *et al.*, 2016), *discover_mutex*, ranked first across all BRCA models (Supplementary Fig. S17). Gene co-expression in healthy tissue samples (GTEx), *gtex_corr* and co-expression in matched normal tissue samples from cancer patients (TCGA), *normal_corr*, respectively, ranked second and third for all BRCA models (Supplementary Fig. S17). Differential expression features, *diff_exp_logFC* and *diff_exp_pvalue*, were most important for LUAD random forest models (Supplementary Fig. S18). These results indicate that features other than those based on gene dependency could also be informative for SL prediction.

## 4 Conclusion

We proposed SL prediction models with increased resilience to selection bias (SBSL models). We used logistic regression and random forest models based on molecular features characterizing genes and gene pair relationships. Without explicit knowledge of gene or pair identity, SBSL models generalized better across SL label datasets and were more robust to gene holdout compared to methods that predict based on the structure of SL labels. In addition, SBSL models improved over existing feature-based SL prediction approaches by focusing on underexplored data such as cancer cell line gene dependencies with mutation data, gene expression from healthy donors, mutual exclusivity of somatic mutations in patient tumours and cancer patient survival. One limitation of our SBSL models is that they rely heavily on gene dependency scores, which are not available for rarer cancer types. We showed that other features could partially compensate for the absence of gene dependency scores, but led to a significant decrease in performance. In addition, we also note that some of the most relevant features in SBSL models are less effective for cancer types typically characterized by low mutational burden. Further research is therefore needed into alternative data and strategies to improve SL prediction. Since the ultimate goal of SL prediction models is to identify SL partners for drug target genes, systematic validation of SBSL models should be conducted to assess the therapeutic potential of predicted pairs.

Analysis of SL label data revealed the presence of strong gene selection bias. Further experiments showed that SL prediction methods relying on the structure of SL labels were more sensitive to such bias. This vulnerability persisted even when the methods incorporated additional data sources. Our observations align with a study on the prediction of protein–protein interactions by Richoux *et al.* (2019), which showed that including the same proteins in the train and test set led to performance overestimation. We believe that performances reported for SL topology methods under these conditions could be optimistic and should be viewed with caution.

We put forward two recommendations for the evaluation of SL prediction models. First, inspecting performance across cancer types, SL datasets and other variables of interest is crucial to ensure that results are consistent and reproducible. Second, we advocate that gene selection biases are considered to avoid that performance metrics report on ability to exploit selection bias rather than predict SL interactions. We show that plotting SL label adjacencies and conducting gene holdout experiments are effective ways to assess selection bias and its impact on SL prediction.

## Supplementary Material

btac523_Supplementary_Data

## Data Availability

The data underlying this article were derived from sources in the public domain. All sources are detailed in the Materials and Methods section.
