# Supplementary material for: Overcoming selection bias in synthetic lethality prediction
Source: Bioinformatics. 2022 Jul 25;38(18):4360–8. doi: 10.1093/bioinformatics/btac523 (PMC9477536; doi:10.1093/bioinformatics/btac523)
Supplement: btac523_Supplementary_Data [file btac523_supplementary_data.pdf]

Overcoming Bias in Synthetic Lethality Prediction
Supplementary Material

Colm Seale, Yasin Tepeli, Joana P. Gonçalves

Pattern Recognition & Bioinformatics, Department of Intelligent Systems, EEMCS Faculty
Delft University of Technology, The Netherlands

Contents

Supplementary Methods **2**
SL methods for comparison (DAISY, DiscoverSL, pca-gCMF, GRSMF, GCATSL) . . . 2

List of Tables

S1 Features used in SBSL prediction models. . . . 3
S2 Performance of one-cancer and pan-cancer SL prediction models - AUROC. . . . 4
S3 Performance of one-cancer and pan-cancer SL prediction models - AUPRC. . . . 5
S4 Feature variance inflation factors. . . . 6
S5 Functional associations found for the top 5 ranked SBSL-L0L2 predictions for BRCA and LUAD. . . . 7

List of Figures

S1 Hyperparameter search for the L0L2 model. . . . 8
S2 Hyperparameter search for the ElasticNet model. . . . 9
S3 Hyperparameter search for the RRF model. . . . 10
S4 Average receiver-operating characteristic (ROC) curves and precision-recall (PR) curves. . . . 11
S5 Structure of BRCA SL labels . . . 12
S6 Structure of COAD SL labels . . . 13
S7 Structure of LUAD SL labels . . . 14
S8 Structure of OV SL labels . . . 15
S9 Cross-SL gold standard performances - AUPRC. . . . 16
S10 Structure of BRCA SL labels in ISLE and DiscoverSL labels. . . . 17
S11 Structure of LUAD SL labels in ISLE and DiscoverSL labels. . . . 18
S12 Gene holdout performances - AUPRC. . . . 19
S13 Cross-cancer performance - AUROC. . . . 20
S14 Cross-cancer performance - AUPRC. . . . 21
S15 Feature importance for BRCA models. . . . 22
S16 Feature importance for LUAD models. . . . 23
S17 Feature importance for BRCA models without gene dependency-based features. . . . 24
S18 Feature importance for LUAD models without gene dependency-based features. . . . 25

Supplementary methods

Synthetic lethality prediction methods for comparison

DAISY

DAISY is a statistical method that consists of three statistical tests. The first, "Genomic Survival of the Fittest" (GSOF) is defined as the p-value of a two-tailed Wilcoxon rank-sum test measuring the change in somatic copy number alteration (SCNA) for gene A between cell lines with an "inactivated" gene B, and those without (an "inactivation" of a gene in a cell line is defined as a gene possessing a non-silent mutation or an SNCA score of < -0.3). The second test calculates the significance of the difference in RNAi-based gene dependency scores between the cell lines, under the same conditions as for GSOF. Lastly, the significance of the Pearson's correlation between the expression of two genes across samples is calculated. These three p-values are then combined using Fisher's method. We conducted the GSOF and Pearson's correlation tests across cancer cell line and patient tumour datasets and combined the p-values using Fisher's method (see "Extended Experimental Procedures" provided in the supplementary materials of [16]). The RNAi-based gene dependency tests were limited to only cancer cell line data.

DiscoverSL

DiscoverSL uses a random forest model trained on breast and lung cancer data [6]. The DiscoverSL model incorporates four features: differential expression and expression correlation in tumour samples, pathway co-participation, and combined mutual exclusivity p-value (see Methods, Section 2.2). The pre-trained model was made public as an R package for classification of SL pairs. We used this pre-trained model in our experiments.

PCA-gCMF

The pca-gCMF method uses a combination of principal component analysis (PCA) and group-sparse collective matrix factorisation (gCMF) to predict SL interactions [21]. Group-space collective matrix factorisation decomposes an arbitrary collection of matrices into lower-rank matrices, while imposing a group-sparse penalty on these decomposed low-rank matrices [17]. PCA transformations are used as a method to overcome a gCMF limitation whereby unique representations for each entity cannot be learned when multiple input matrices contain identical row and column entity-types. We used pca-gCMF with four feature matrices as presented in their paper:

- *Gene dependency profiles.* A $p \times p$ matrix with p genes. For any gene pair (A, B), the p-value of a Wilcoxon rank sum test is calculated which quantifies the change dependency scores for gene A for cell-lines with and without a mutation in gene B. This test is conducted in reverse on (B,A) and the smallest p-value is used in the resulting matrix.
- *mRNA expression profiles:* A $p \times q$ matrix with p genes and q patient tumour samples, where each element of the matrix is the mRNA expression count.
- *Co-expression:* A $p \times q$ matrice with p genes where the p-value for the Spearman correlation coefficient is measured between gene pairs across all patient tumour samples.
- *CNV profile:* A $p \times q$ matrice with p genes and q patient tumour samples, where each element of the matrice are the CNV real-valued scores produced by the GISTIC algorithm.

GRSMF

Graph regularized self-representative matrix factorisation (GRSMF) is a matrix factorisation method which learns self-representations from the existing SL labels, and regularises these representations using similarities derived from Gene Onotology [14](Downloaded on 18 March 2021 from `http://purl.obolibrary.org/obo/go/go-basic.obo`). We created the GO similarity matrix as described by the authors. We use the best hyperparameters as provided by the authors.

GCATSL

GCATSL uses contextualised attention networks to find synthetic lethal relations [24]. It calculates three different similarity matrices using GO Biological Process, GO Cellular Component and PPI graph. In our implementation, we derived GO data from Gene Ontology[1](Downloaded on 18 March 2021 from `http://purl.obolibrary.org/obo/go/go-basic.obo`) and we use STRING [35](Version 11.0 from `https://stringdb-static.org/download/protein.links.v11.0/9606.protein.links.v11.0.txt.gz`) as our PPI source instead of BioGrid as originally used by the authors [34]. We use the best hyperparameters as provided by the authors.

Supplementary Tables

Table S1: Features used in SBSL prediction models. Columns "Biological sample" and "Data type" indicate the type of biological samples and the kind of data (e.g. molecular profiles) acquired for those samples, respectively. Descriptions of how each feature was calculated, and sources of the data, are provided in the main manuscript (Methods). The "Used" column indicates where this specific feature was first used in the context of computational SL prediction.

Symbol	Description	Biological sample	Data type	Used
CRISPR_dep_stat	Change in CRISPR dependency score of one gene based on non-silent mutations in the other (Wilcoxon)	Cancer cell lines	CRISPR dependency [29, 7] Mutation [12]	Here
CRISPR_dep_pvalue	Significance of change in dependency of one gene based on non-silent mutations in the other (Wilcoxon)	Cancer cell lines	CRISPR dependency [29, 7] Mutation [12]	Here
CRISPR_cor_stat	Correlation of gene-wise CRISPR dependency scores (Pearson's)	Cancer cell lines	CRISPR dependency [29, 7]	Here
CRISPR_cor_pvalue	Significance of correlation of gene-wise CRISPR dependencies (t-test)	Cancer cell lines	CRISPR dependency [29, 7]	Here
CRISPR_avg	Average of gene-wise means of CRISPR dependency scores	Cancer cell lines	CRISPR dependency [29, 7]	Here
RNAi_dep_stat	See CRISPR equivalent	Cancer cell lines	RNAi dependency [36, 27] Mutation [12]	Here
RNAi_dep_pvalue	See CRISPR equivalent	Cancer cell lines	RNAi dependency [36, 27] Mutation [12]	[16]
RNAi_cor_stat	See CRISPR equivalent	Cancer cell lines	RNAi dependency [36, 27]	Here
RNAi_cor_pvalue	See CRISPR equivalent	Cancer cell lines	RNAi dependency [36, 27]	Here
RNAi_avg	See CRISPR equivalent	Cancer cell lines	RNAi dependency [36, 27]	Here
discover_mutex	Mutual exclusivity score [4]	Patient tumour	CNV, mutation [28, 19]	Here
discoversl_mutex_amp	Significance of non-co-occurrence of amplifications (hypergeom.)	Patient tumour	CNV [28, 19]	[6]
discoversl_mutex_del	Significance of non-co-occurrence of deletions (hypergeom.)	Patient tumour	CNV [28, 19]	[6]
discoversl_mutex_mut	Significance of non-co-occurrence of non-silent mutations (hypergeom.)	Patient tumour	Mutation [19]	[6]
discoversl_mutex	Combined p-value of previous three scores using Fisher's method	Patient tumour	CNV, mutation [28, 19]	[6]
mutex_alt	Significance of non-co-occurrence of amplifications, deletions or non-silent mutations	Patient tumour	CNV, mutation [28, 19]	Here
MUTEX	Mutual exclusivity score [2] (failed to identify mutually exclusive pairs in our datasets and was thus not used in training)	Patient tumour	Mutation [19]	Here
logrank_pval	Significance of change in survival time [3] between patients with(out) aberrant expression or CNV in both genes	Patient tumour	CNV, mutation, expression, [28, 19, 31] patient clinical data [31]	Here
diff_exp_logFC	Differential expression of a gene based on mutations in other (log fold-change)	Patient tumour	Mutation, expression [19, 31]	[6]
diff_exp_pvalue	Significance of differential expression of a gene based on mutations in the other (edgeR test p-value, [32])	Patient tumour	Mutation, expression [19, 31]	[6]
gtex_corr	Co-expression (Pearson's correlation)	Healthy donor	Expression [25]	Here
gtex_corr._pvalue	Significance of co-expression (t-test)	Healthy donor	Expression [25]	Here
tumour_corr	Co-expression (Pearson's correlation)	Patient tumour	Expression [31]	[6]
tumour_corr._pvalue	Significance of co-expression (t-test)	Patient tumour	Expression [31]	[6]
normal_corr	Co-expression (Pearson's correlation)	Patient normal	Expression [31]	Here
normal_corr._pvalue	Significance of co-expression (t-test)	Patient normal	Expression [31]	Here
pathway_coparticipation	Significance of co-occurrence in pathways (hypergeom.)	Pathway databases	Pathway gene sets [22]	[6]

Table S2: Performance of one-cancer and pan-cancer SL prediction models (with unbalanced and balanced cancer representation) tested on heldout examples of each cancer type. Mean and standard deviation of AUROC for 10 repetitions.

	Pan-cancer		Cancer-specific
	Unbalanced	Balanced	
BRCA	.64 ± .02	.75 ± .01	**.83 ± .01**
COAD	.52 ± .02	.51 ± .02	**.60 ± .02**
LUAD	.73 ± .03	.79 ± .02	**.83 ± .02**
OV	.40 ± .04	.53 ± .04	**.58 ± .03**

(a) L0L2

BRCA	.65 ± .02	.77 ± .02	**.84 ± .01**
COAD	.52 ± .02	.53 ± .02	**.60 ± .02**
LUAD	.74 ± .02	.80 ± .02	**.85 ± .02**
OV	.40 ± .04	.50 ± .04	**.59 ± .03**

(b) Elastic Net

BRCA	.76 ± .01	.82 ± .02	**.86 ± .01**
COAD	.62 ± .02	.60 ± .01	**.64 ± .01**
LUAD	.81 ± .02	.83 ± .02	**.86 ± .01**
OV	**.55 ± .06**	.52 ± .04	.54 ± .07

(c) MUVR

BRCA	.75 ± .02	.80 ± .02	**.86 ± .01**
COAD	.62 ± .02	.61 ± .02	**.63 ± .02**
LUAD	.80 ± .02	.83 ± .02	**.87 ± .02**
OV	.55 ± .04	.53 ± .05	**.57 ± .07**

(d) RRF

Table S3: Performance of one-cancer and pan-cancer SL prediction models (with unbalanced and balanced cancer representation) tested on heldout examples of each cancer type. Mean and standard deviation of AUPRC for 10 repetitions.

	Pan-cancer		Cancer-specific
	Unbalanced	Balanced	
BRCA	$.68 \pm .02$	$.78 \pm .01$	$\mathbf{.88 \pm .01}$
COAD	$.53 \pm .02$	$.52 \pm .02$	$\mathbf{.59 \pm .02}$
LUAD	$.78 \pm .02$	$.84 \pm .01$	$\mathbf{.87 \pm .02}$
OV	$.46 \pm .02$	$.51 \pm .03$	$\mathbf{.58 \pm .05}$

(a) L0L2

BRCA	$.68 \pm .02$	$.81 \pm .03$	$\mathbf{.87 \pm .01}$
COAD	$.53 \pm .02$	$.52 \pm .02$	$\mathbf{.59 \pm .01}$
LUAD	$.79 \pm .02$	$.83 \pm .02$	$\mathbf{.87 \pm .02}$
OV	$.45 \pm .03$	$.50 \pm .02$	$\mathbf{.58 \pm .04}$

(b) Elastic Net

BRCA	$.82 \pm .01$	$.85 \pm .01$	$\mathbf{.89 \pm .01}$
COAD	$.61 \pm .03$	$.60 \pm .02$	$\mathbf{.62 \pm .01}$
LUAD	$.84 \pm .03$	$.84 \pm .02$	$\mathbf{.87 \pm .01}$
OV	$\mathbf{.54 \pm .05}$	$.52 \pm .05$	$.51 \pm .05$

(c) MUVR

BRCA	$.81 \pm .01$	$.84 \pm .02$	$\mathbf{.89 \pm .01}$
COAD	$.60 \pm .02$	$.60 \pm .03$	$\mathbf{.63 \pm .02}$
LUAD	$.81 \pm .03$	$.83 \pm .02$	$\mathbf{.87 \pm .02}$
OV	$\mathbf{.55 \pm .05}$	$.52 \pm .05$	$.54 \pm .05$

(d) RRF

Table S4: Variance inflation factors (VIF) [15] per feature for the combined dataset. The variance inflation factor indicates how many times higher the variance of the feature coefficient is than one would expect if there was no collinearity. A VIF of 1 indicates that a feature does not correlate with any other features. A VIF of 2 indicates that the variance of a particular feature coefficient is two times higher than one would expect if there was no collinearity, indicating moderate correlation with other features. A VIF value larger than 5 is considered to indicate high correlation with other features.

Feature	VIF
discoversl_mutex_amp	1.388619555
discover_mutex	1.039665442
mutex_alt	1.381683071
RNAi_avg	2.224074149
RNAi_cor_pvalue	1.011676996
RNAi_cor_stat	1.013070426
RNAi_dep_pvalue	1.109236607
RNAi_dep_stat	4.917394579
CRISPR_avg	2.247942347
CRISPR_cor_pvalue	1.007564922
CRISPR_cor_stat	1.006988081
CRISPR_dep_pvalue	1.141351776
CRISPR_dep_stat	4.953596929
gtex_corr	1.334004715
gtex_corr.pvalue	1.071894916
tumour_corr	1.170333149
tumour_corr.pvalue	1.048525217
normal_corr	1.289831761
normal_corr.pvalue	1.430066751
diff_exp_logFC	1.023954926
diff_exp_pvalue	1.072218472
pathway_coparticipation	1.018279983
logrank_pvals	1.347337851

Table S5: Functional associations found for the top 5 ranked SBSL-L0L2 predictions for BRCA and LUAD. As evidence for functional associations specific to the cancer type in question was rarely available, we instead report functional associations in general. We report the top 5 ranked SL pairs as predicted by the L0L2 model, as the SBSL linear models generalised better across SL gold standard datasets and cancer types. There were no significant differences between the predictions of the L0L2 and Elastic Net models.

Cancer Type	Gene Pair	Functional Association
BRCA	TP53/BOP1	BOP1 is a regulator of p53 pathway function. [30]
	TP53/CRYGS	CRYGS is a target gene of the TP53 transcription factor. [18]
	TP53/EEF1D	Not found.
	TP53/AKAP8L	Physical interaction between AKAP8L and TP53 proteins. [23]
	TP53/CPSF1	Not found.
LUAD	KRAS/ANAPC4	ANAPC4 is synthetic lethal with KRAS. [26]
	KRAS/FBL	Not found.
	KRAS/PSMA1	PSMA1 is synthetic lethal with KRAS. [5]
	KRAS/PSMA5	Not found.
	KRAS/RRM2	KRAS is a regulator of RRM2. [37]

Supplementary Figures

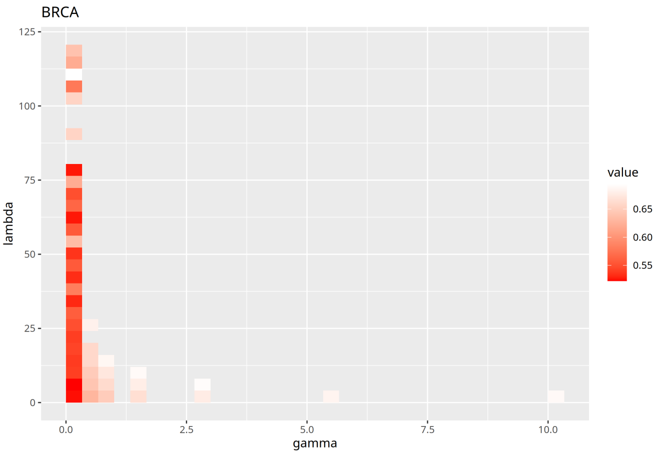

(a) BRCA

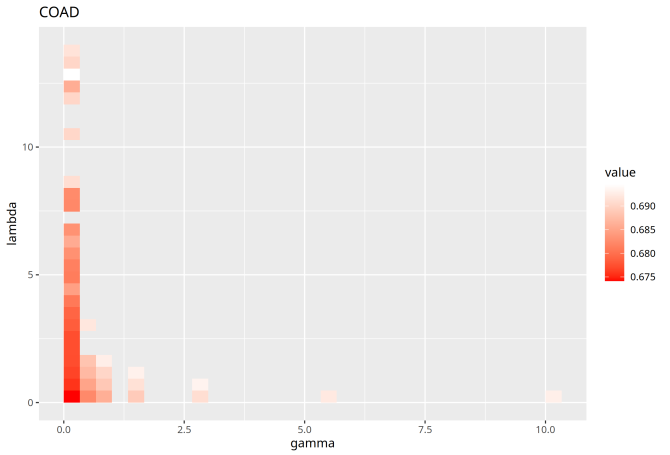

(b) COAD

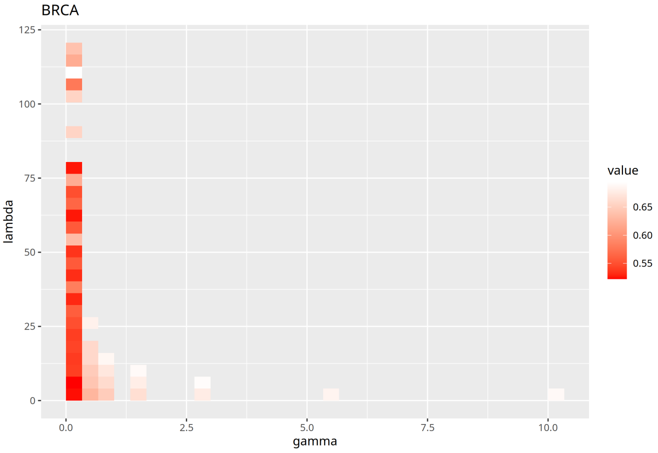

(c) LUAD

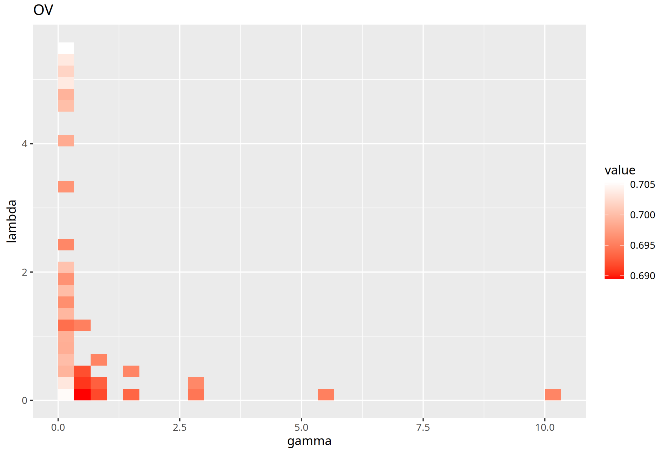

(d) OV

Figure S1: Hyperparameter search for the L0L2 model [13]. Mean logistic loss values of the optimised local search cross-validation results across each of the 10 folds across all 10 cross-validation runs for each cancer type. Deeper red values indicate lower mean logistic loss for that combination of *gamma* and *lambda*. L0Learn uses a custom local search algorithm to find the optimal values for *lambda*.

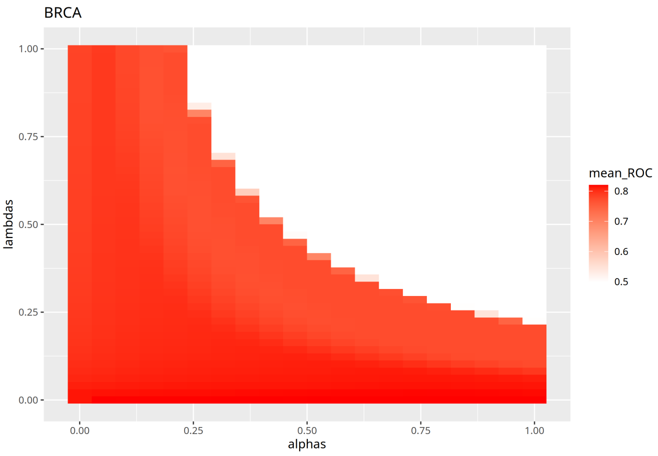

(a) BRCA

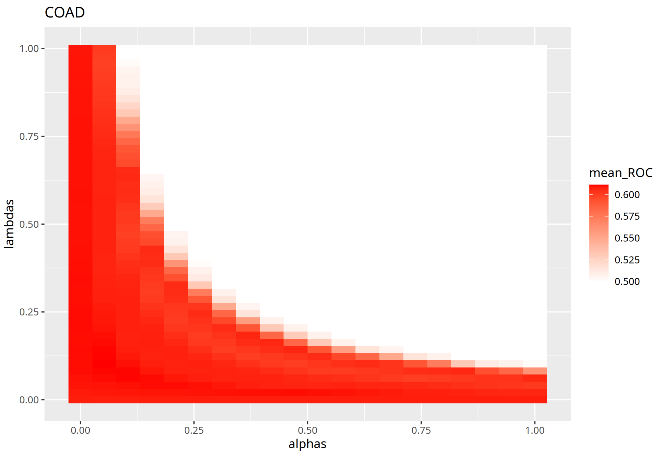

(b) COAD

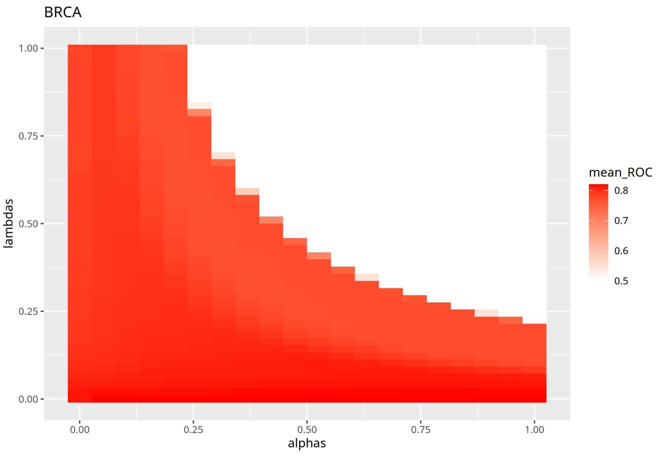

(c) LUAD

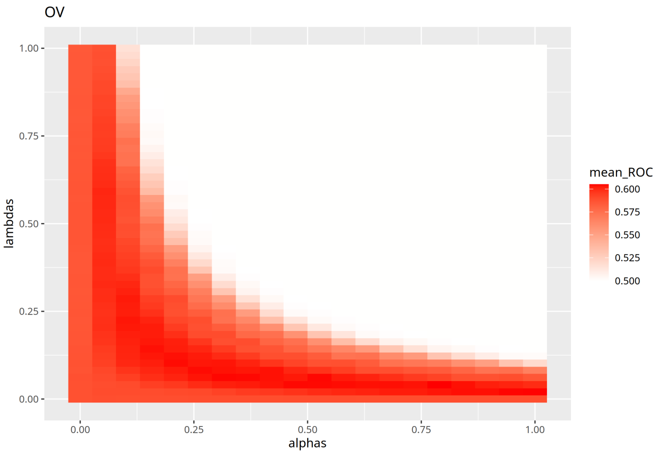

(d) OV

Figure S2: Hyperparameter search for the ElasticNet model [11]. Mean AUROC grid search cross-validation results across each of the 10 folds across all 10 cross-validation runs for each cancer type. Deeper red values indicate higher mean AUROC for that combination of *alpha* and *lambda*.

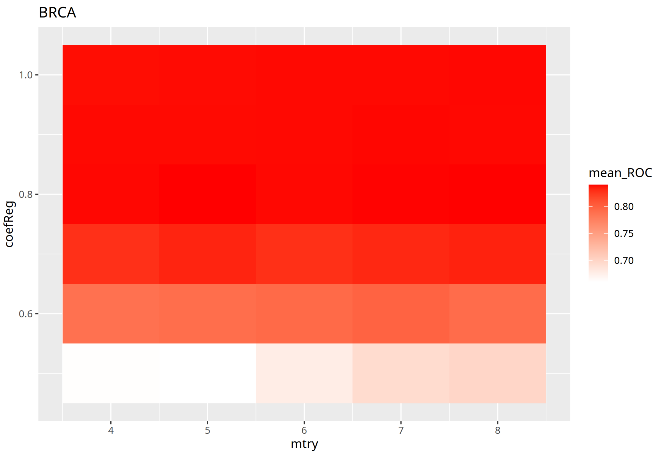

(a) BRCA

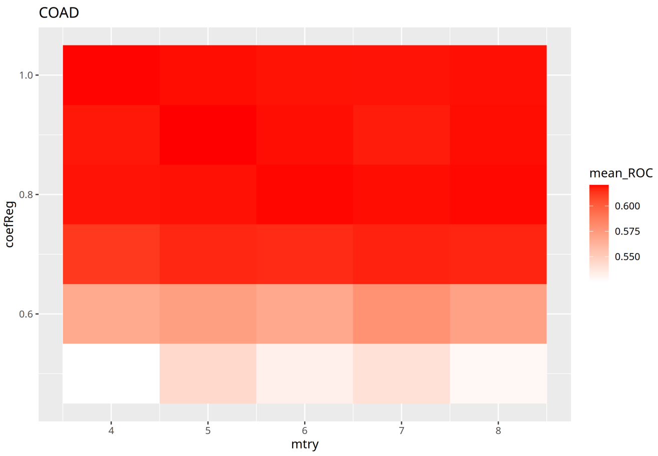

(b) COAD

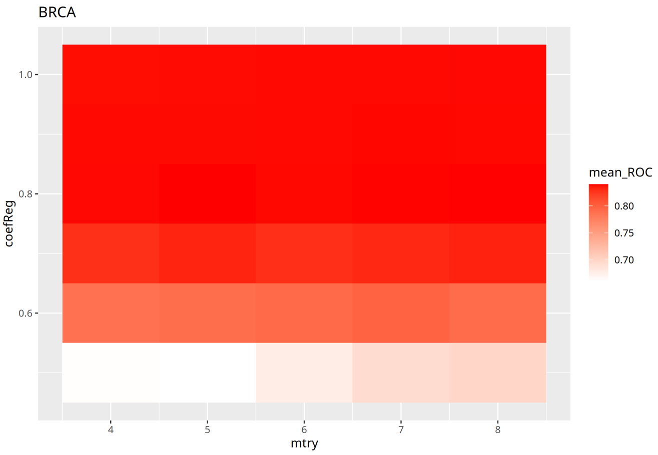

(c) LUAD

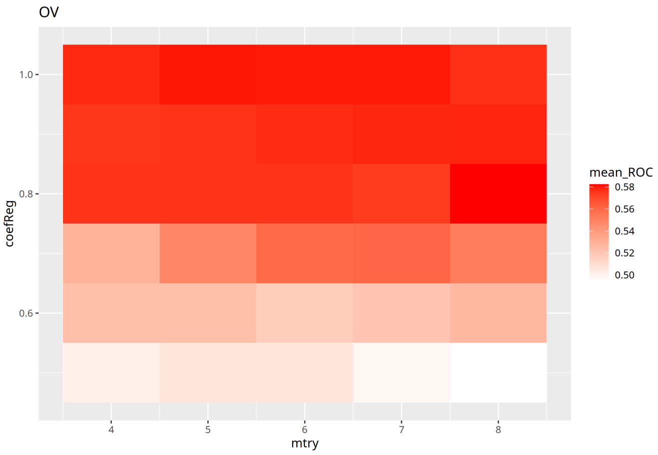

(d) OV

Figure S3: Hyperparameter search for the RRF model [8]. Mean AUROC grid search cross-validation results across each of the 10 folds across all 10 cross-validation runs for each cancer type. Deeper red values indicate higher mean AUROC for that combination of *coefReg* and *mtry*.

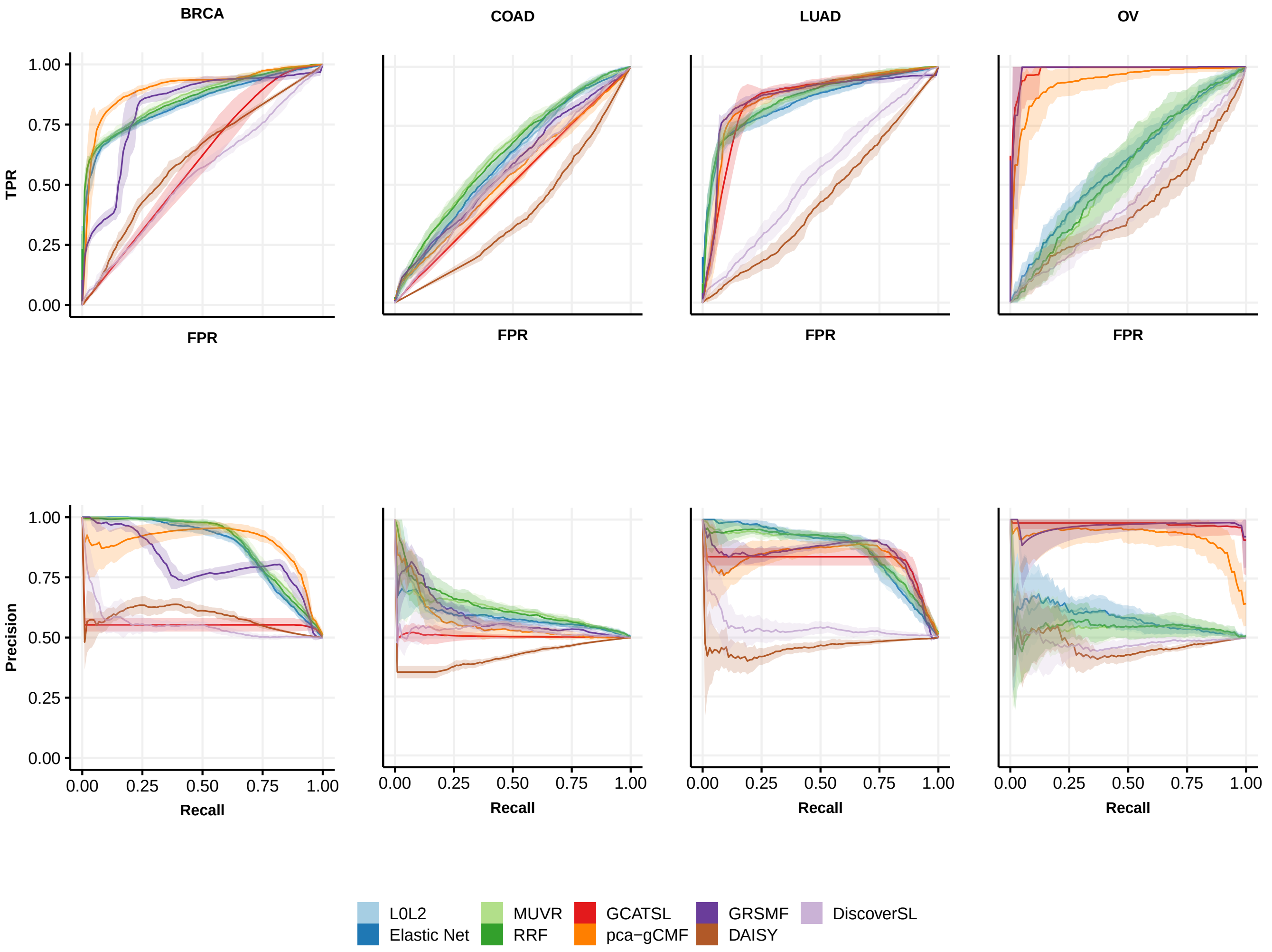

Figure S4: Average receiver-operating characteristic (ROC) curves and precision-recall (PR) curves for each cancer-specific model tested against that same cancer type. The top plots show ROC curves, the bottom plots show PR curves, and each column corresponds to a different cancer type. The ROC and PR curves were averaged across 10 runs using the vertical-averaging method [9]. The shaded regions represent the standard deviation.

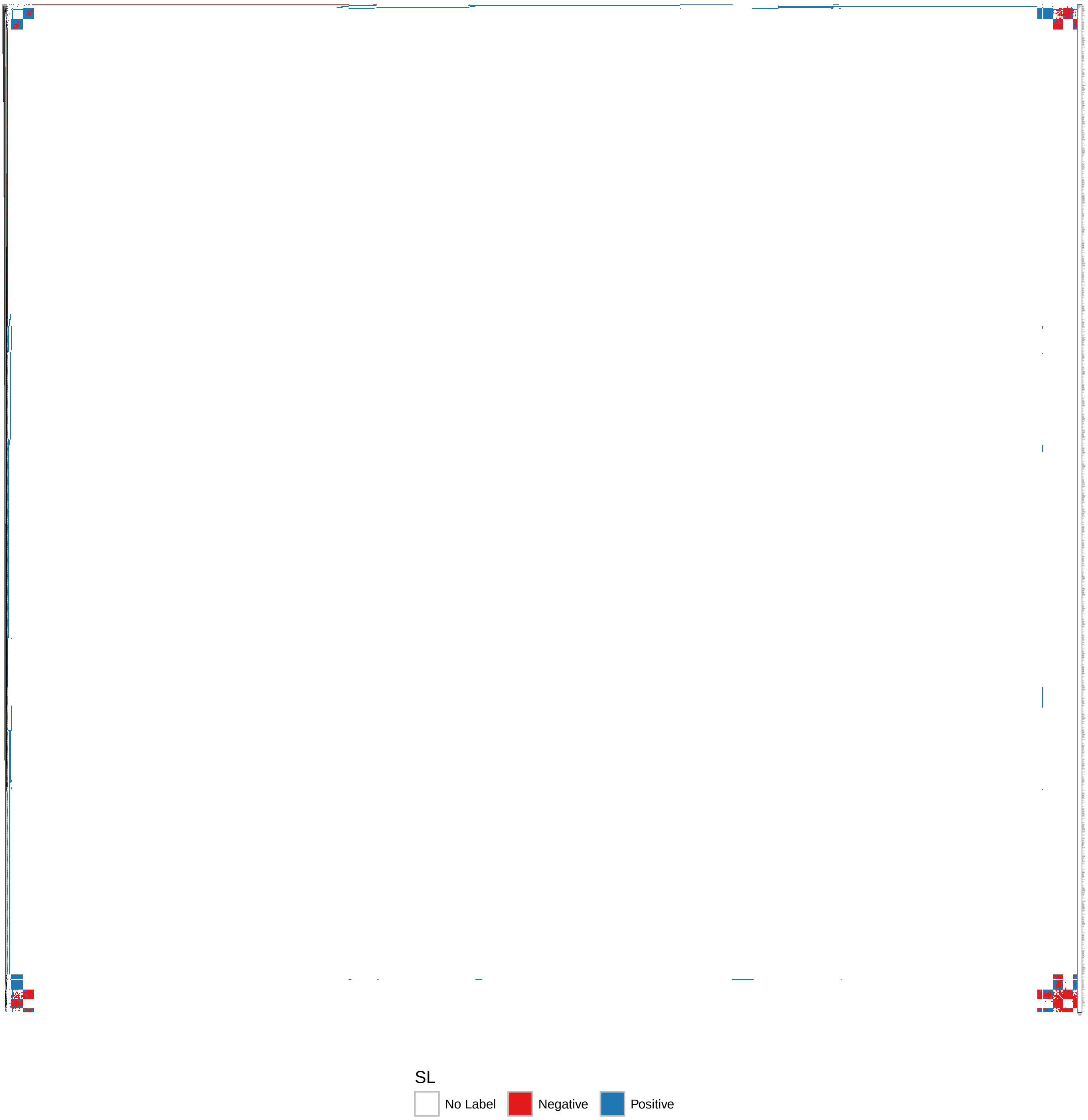

Figure S5: Adjacency matrix plot showing clusters of labelled genes in BRCA. Elements along the horizontal and vertical axes represent unique genes. Each coloured dot corresponds to a negatively (red) or positively (blue) labelled gene pair. Whitespace denotes a gene pair with no label. Rows are clustered using complete linkage and Euclidean distance with “No Label”, “Negative”, and “Positive” encoded as 0.5, 0 and 1, respectively. Both the rows and columns are ordered based on these clusters. The barplot to the right shows the number of occurrences of each gene in the BRCA dataset.

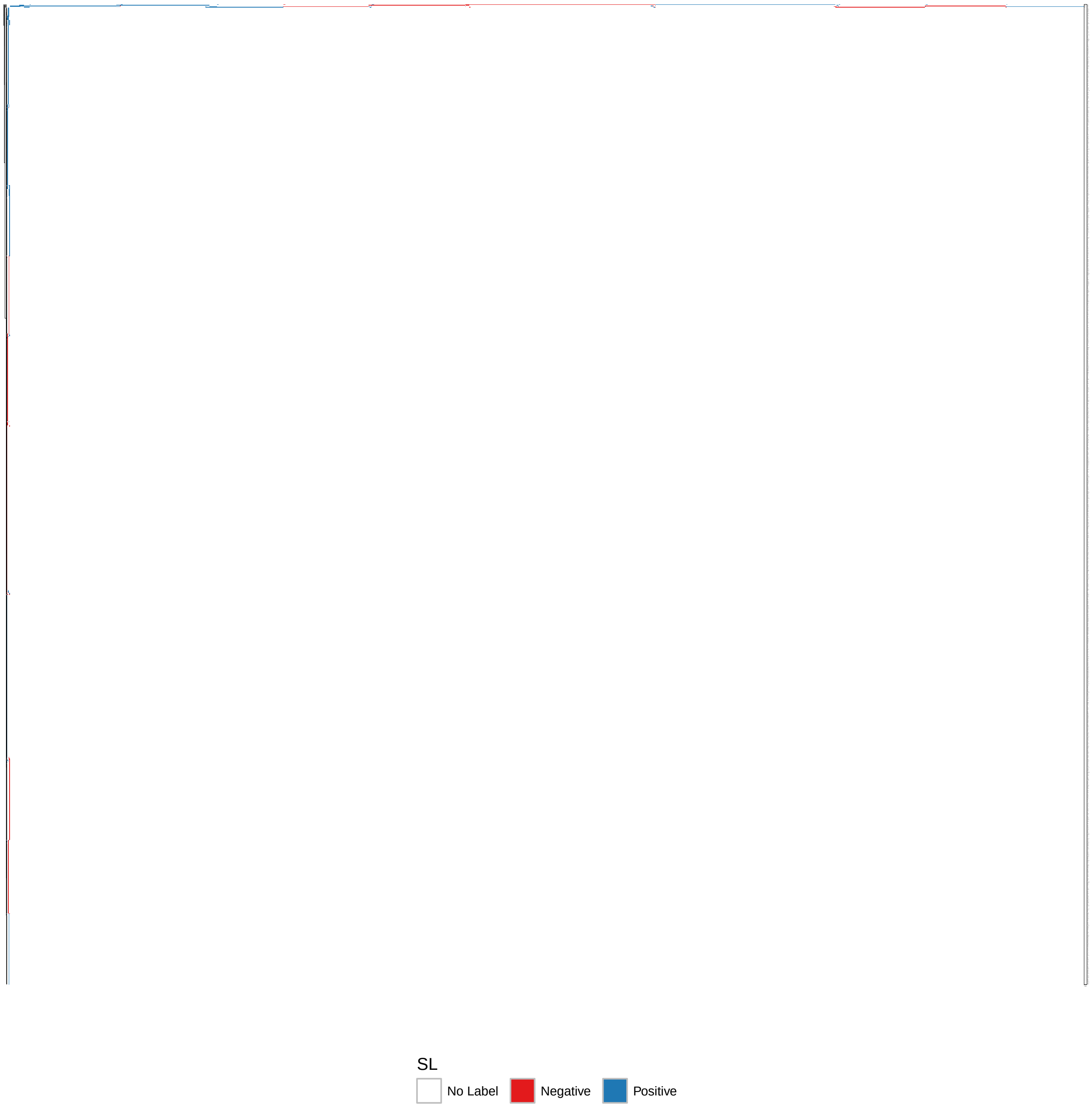

Figure S6: Adjacency matrix plot showing clusters of labelled genes in COAD. Elements along the horizontal and vertical axes represent unique genes. Each coloured dot corresponds to a negatively (red) or positively (blue) labelled gene pair. Whitespace denotes a gene pair with no label. Rows are clustered using complete linkage and Euclidean distance with "No Label", "Negative", and "Positive" encoded as 0.5, 0 and 1, respectively. Both the rows and columns are ordered based on these clusters. The barplot to the right shows the number of occurrences of each gene in the COAD dataset.

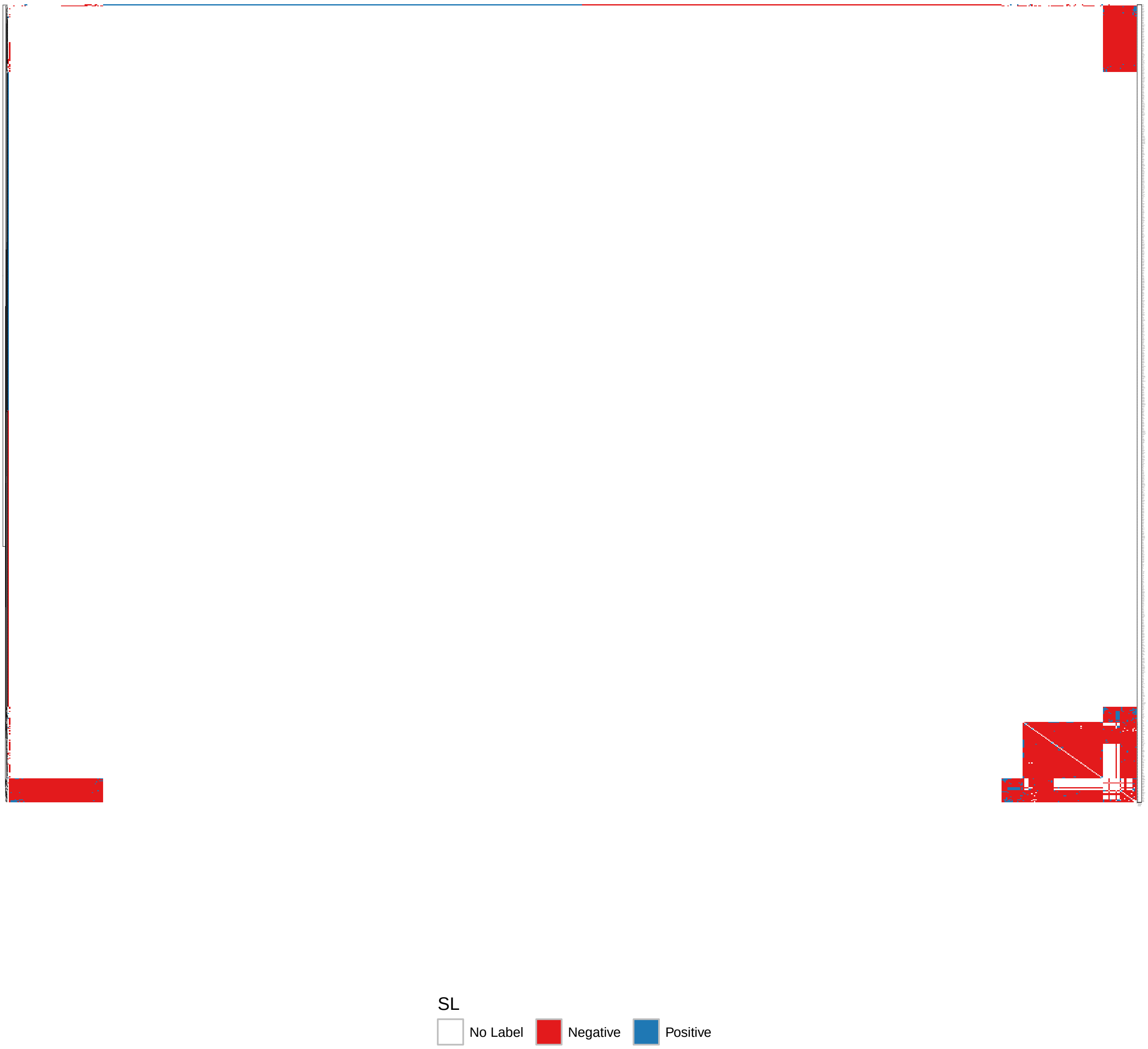

Figure S7: Adjacency matrix plot showing clusters of labelled genes in LUAD. Elements along the horizontal and vertical axes represent unique genes. Each coloured dot corresponds to a negatively (red) or positively (blue) labelled gene pair. Whitespace denotes a gene pair with no label. Rows are clustered using complete linkage and Euclidean distance with “No Label”, “Negative”, and “Positive” encoded as 0.5, 0 and 1, respectively. Both the rows and columns are ordered based on these clusters. The barplot to the right shows the number of occurrences of each gene in the LUAD dataset.

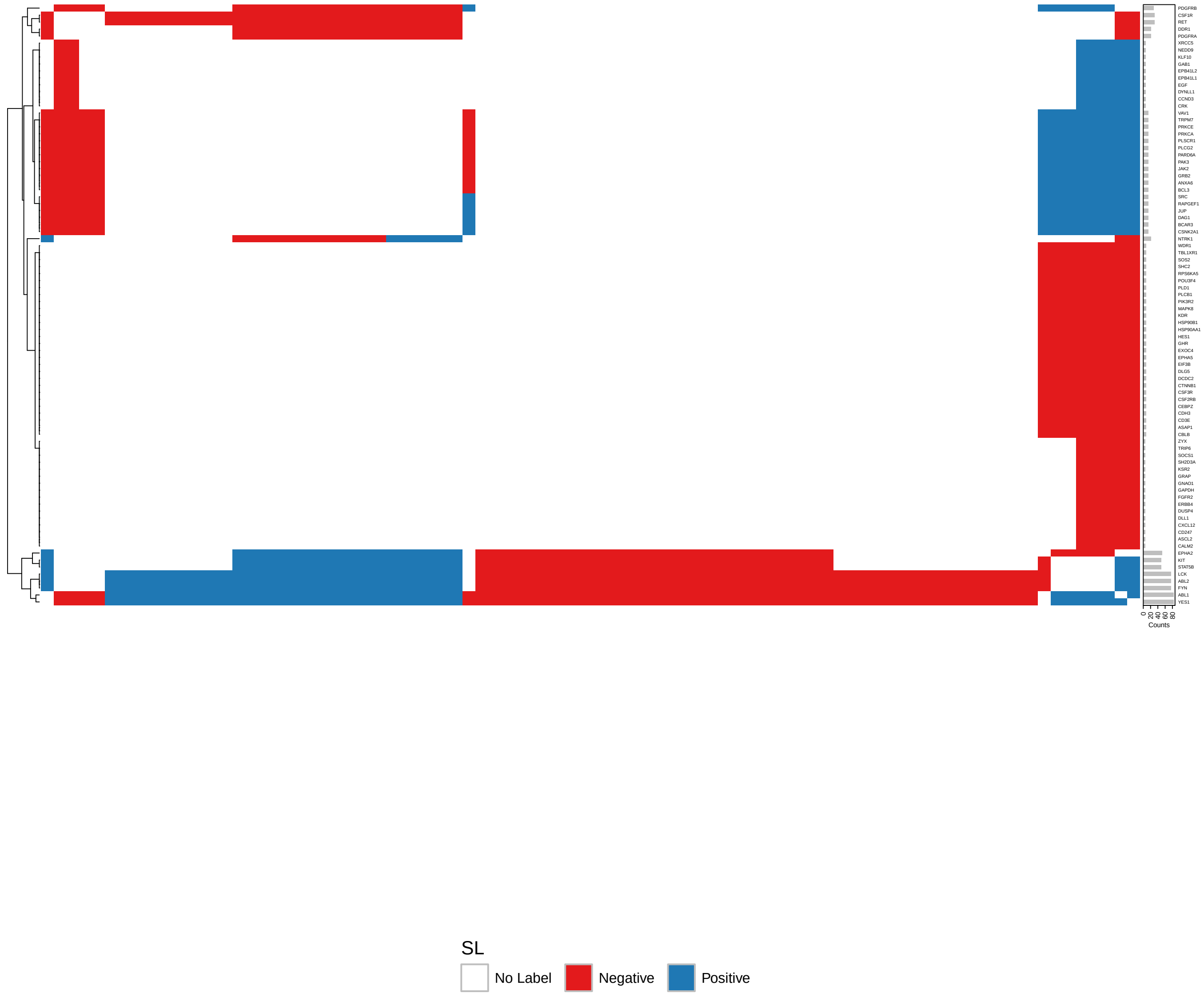

Figure S8: Adjacency matrix plot showing labelled gene pairs in OV. Elements along the horizontal and vertical axes represent unique genes. Each coloured dot corresponds to a negatively (red) or positively (blue) labelled gene pair. Whitespace denotes a gene pair with no label. Rows are clustered using complete linkage and Euclidean distance with “No Label”, “Negative”, and “Positive” encoded as 0.5, 0 and 1, respectively. Both the rows and columns are ordered based on these clusters. The barplot to the right shows the number of occurrences of each gene in the OV dataset.

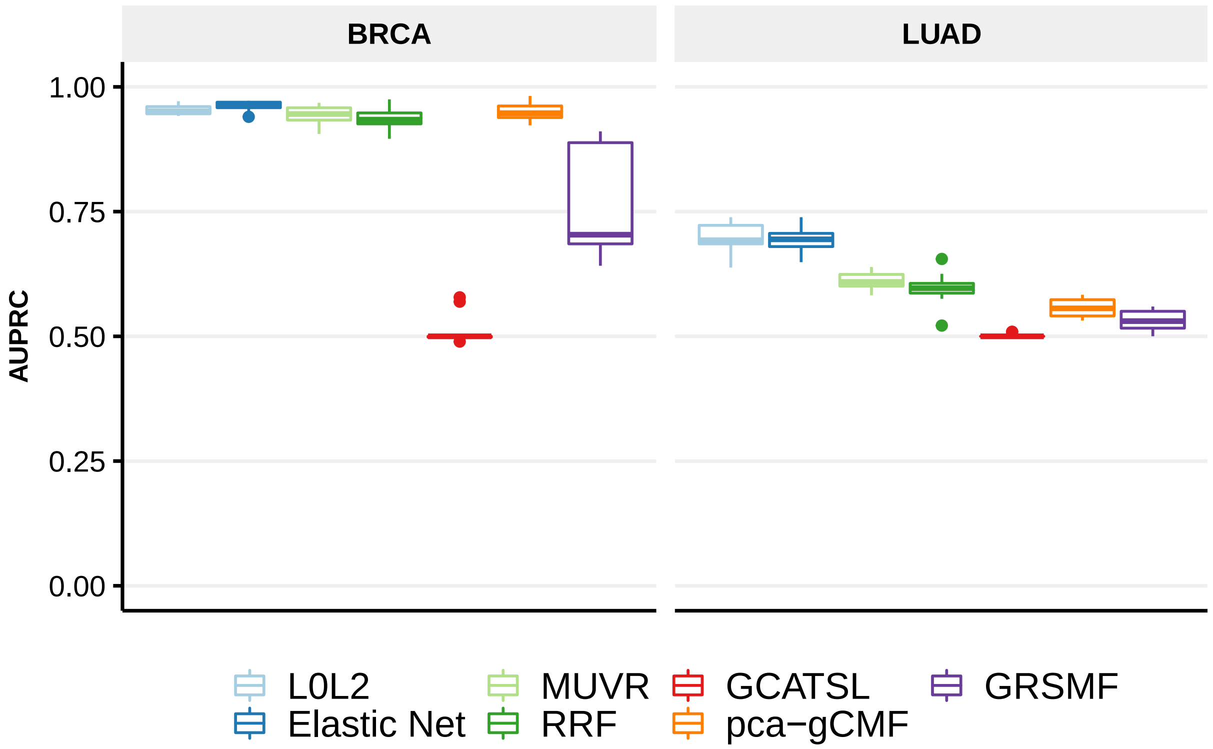

Figure S9: AUPRC values averaged over 10 runs for: (left) BRCA models trained on ISLE and tested on DiscoverSL; (right) LUAD models were trained on DiscoverSL and tested on ISLE.

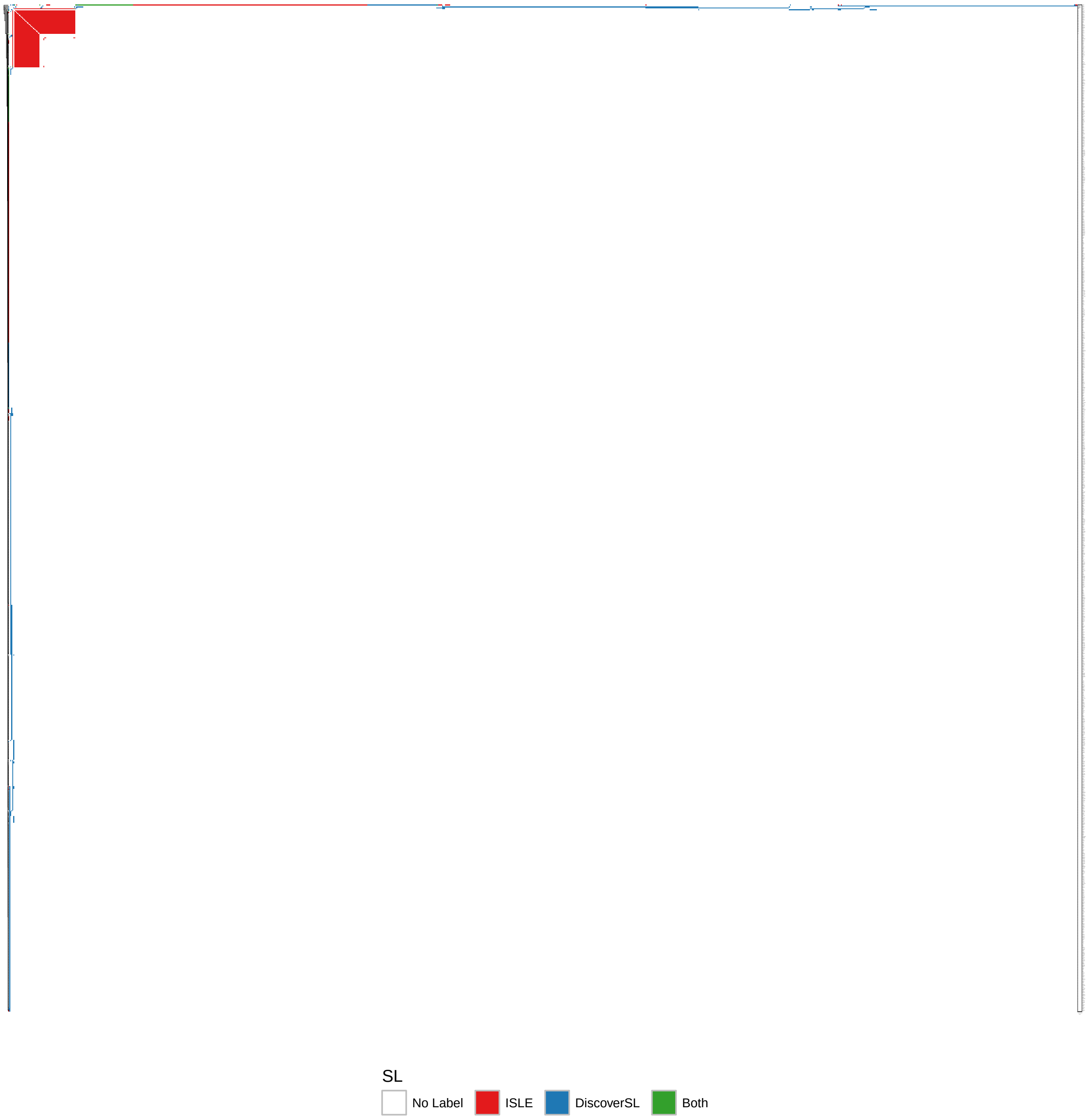

Figure S10: Structure of BRCA SL labels in the ISLE [20] and DiscoverSL datasets [6]. Heatmap showing labelled gene pairs from BRCA data from both the ISLE and DiscoverSL dataset. Elements along the horizontal and vertical axes represent unique genes. Each coloured dot represents a gene pair where a label exists in either the ISLE (red), DiscoverSL (blue), or both (green) datasets. Whitespace denotes a gene pair with no label. Rows are clustered using complete linkage and Euclidean distance with “No Label”, “ISLE”, “DiscoverSL”, and “Both” encoded as -1, 0, 1, and 2, respectively. Both the rows and columns are ordered based on these clusters. The barplot to the right shows the number of occurrences of each gene in the BRCA dataset.

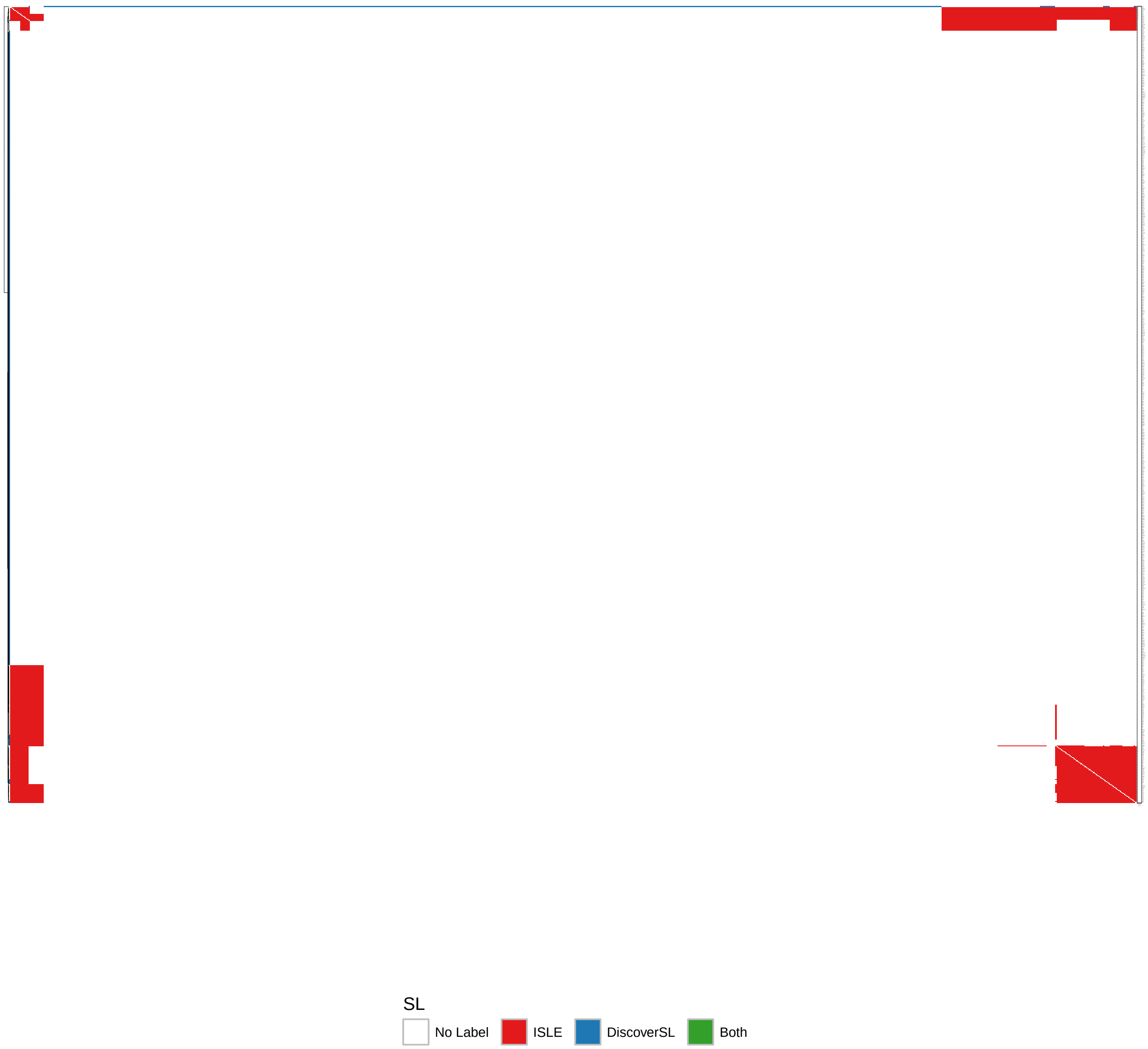

Figure S11: Structure of LUAD SL labels in the ISLE [20] and DiscoverSL datasets [6]. Heatmap showing labelled gene pairs from LUAD data from both the ISLE and DiscoverSL dataset. Elements along the horizontal and vertical axes represent unique genes. Each coloured dot represents a gene pair where a label exists in either the ISLE (red), DiscoverSL (blue), or both (green) datasets. Whitespace denotes a gene pair with no label. Rows are clustered using complete linkage and Euclidean distance with "No Label", "ISLE", "DiscoverSL", and "Both" encoded as -1, 0, 1, and 2, respectively. Both the rows and columns are ordered based on these clusters. The barplot to the right shows the number of occurrences of each gene in the LUAD dataset.

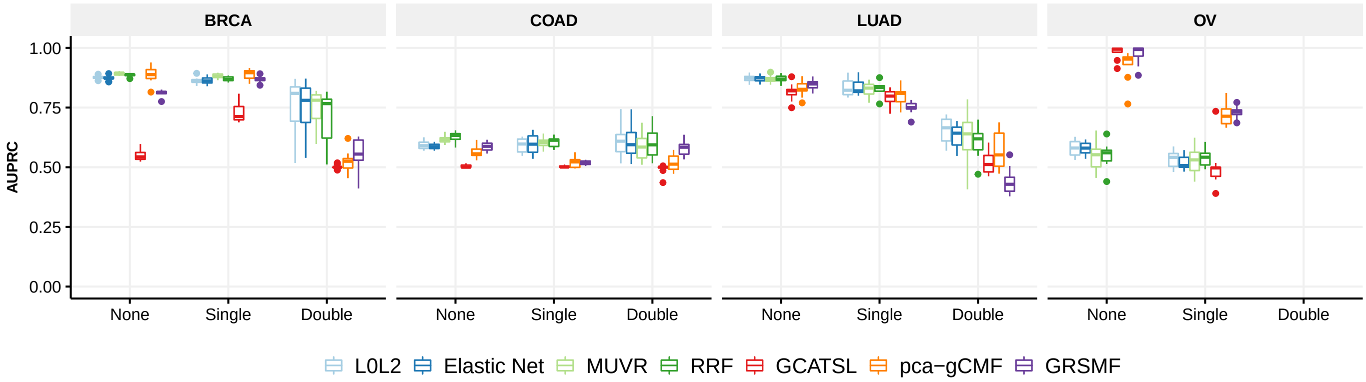

Figure S12: Performances of gene holdout experiments, where bias is controlled by ensuring that none, one, or both genes of pairs in the test set are excluded from the train set. Shown are AUPRC values for each gene-holdout experiment per cancer type (10 runs). For *None*, we only guarantee that train and test sets are disjoint in terms of gene pairs, not individual genes; for *Single*, only one gene from a gene pair in the test set can be present in the train set; for *Double* neither gene of a pair in the test set appears in the train set. The results for "None" are calculated from the same experiment as Tables 2-3 in the main text. Note: there was insufficient data to conduct the OV *Double* experiment.

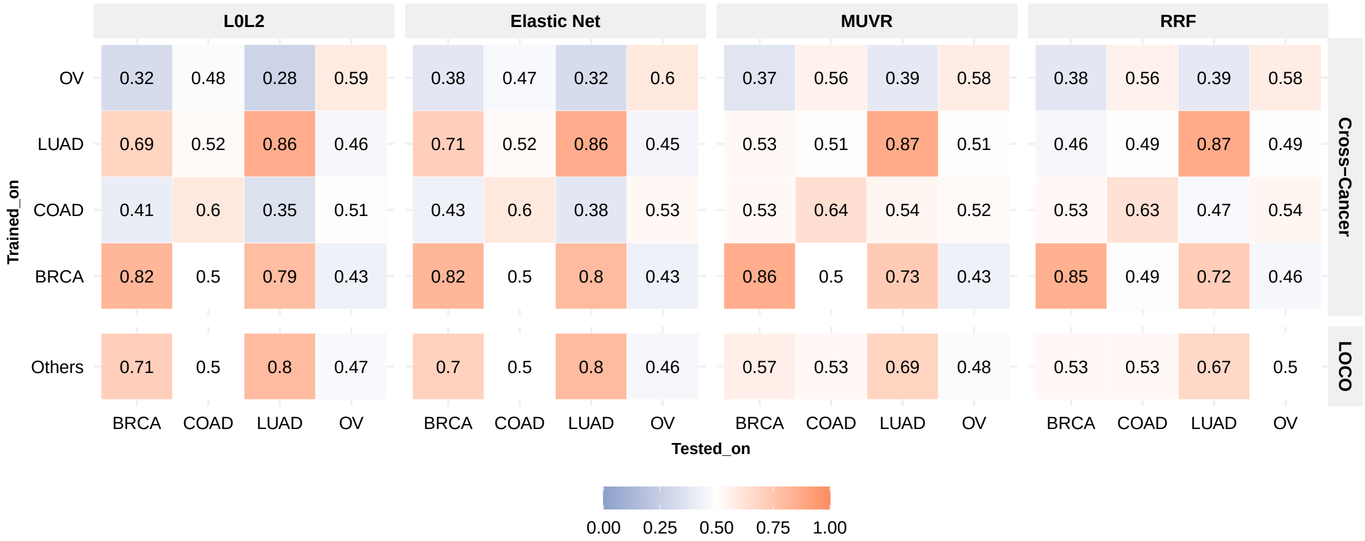

Figure S13: Heatmaps of average AUROC performances for the L0L2 [13], Elastic Net [11], MUVR [33], and RRF [8] models over 10 runs. *Cross-cancer:* Vertical and horizontal axes denote the cancer types used to train and test, respectively. *LOCO:* Horizontal axis denotes the cancer type held out for testing. Models trained on balanced data from all other cancers.

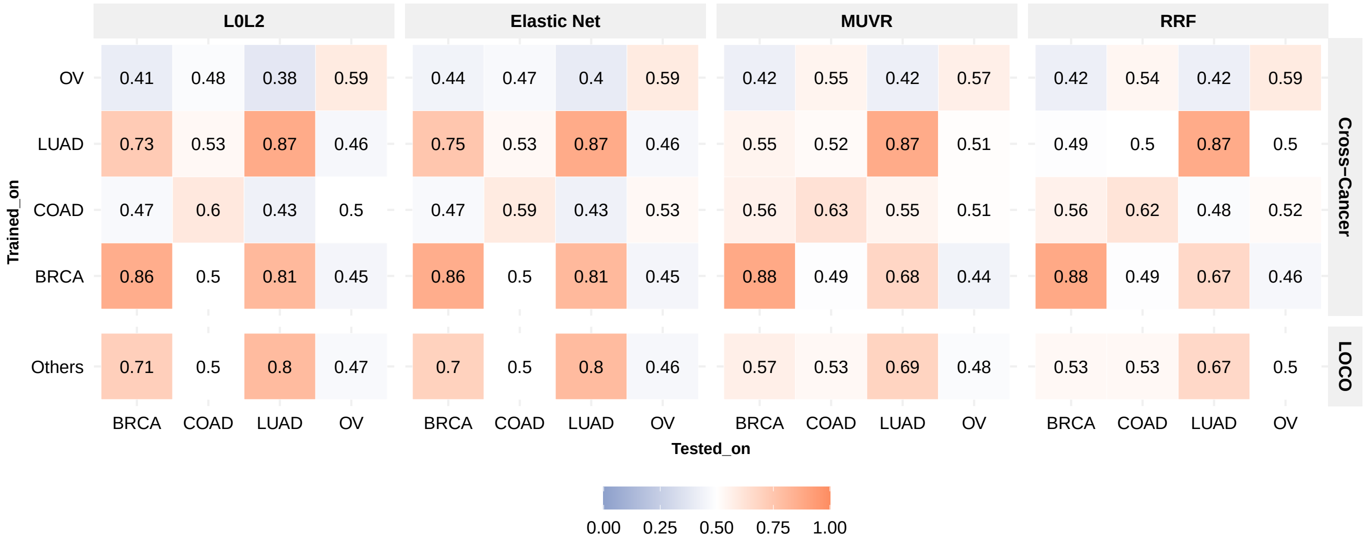

Figure S14: Heatmaps of average AUPRC performances for the L0L2 [13], Elastic Net [11], MUVR [33], and RRF [8] models over 10 runs. *Cross-cancer:* Vertical and horizontal axes denote the cancer types used to train and test, respectively. *LOCO:* Horizontal axis denotes the cancer type held out for testing. Models trained on balanced data from all other cancers.

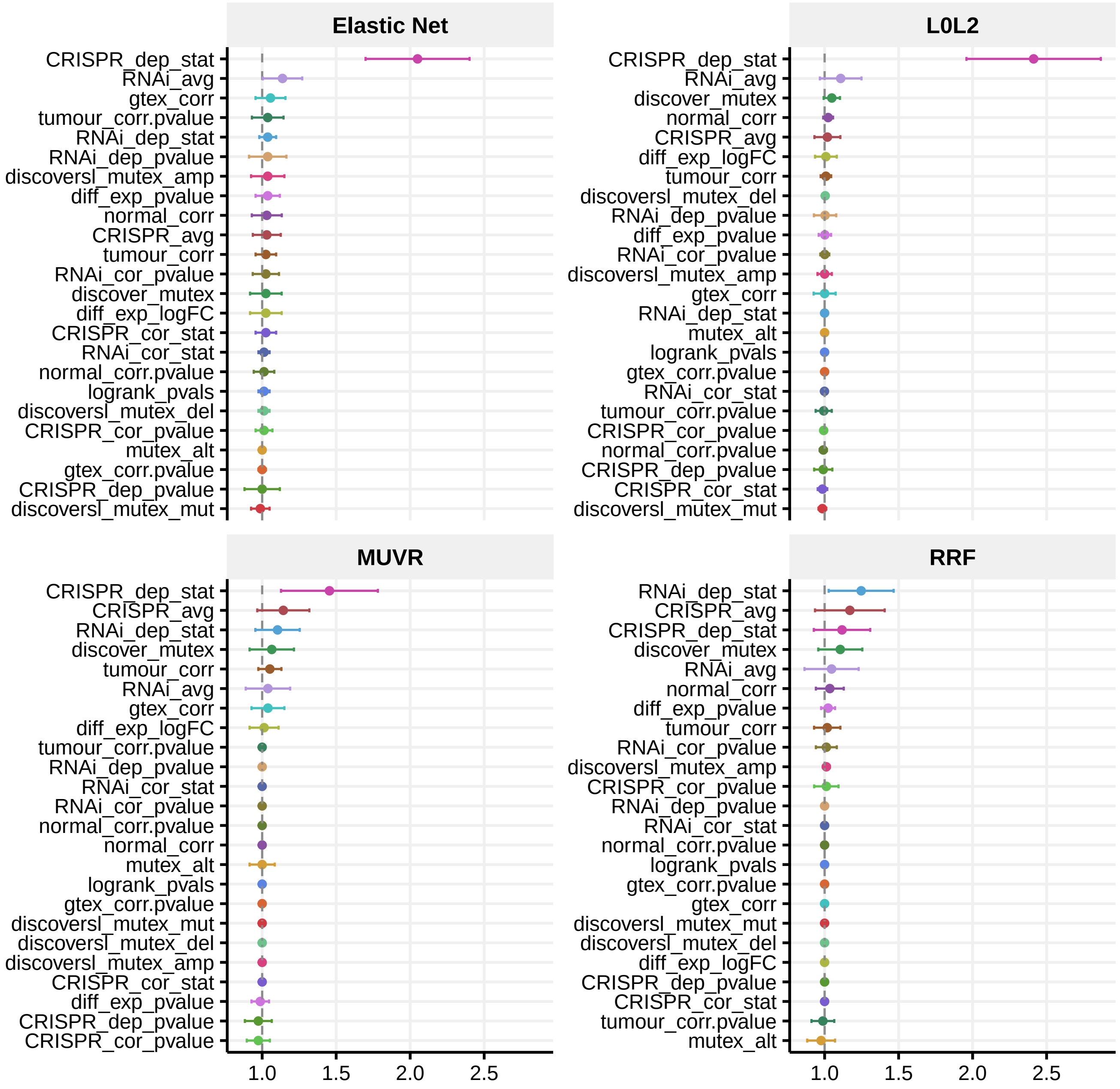

Figure S15: Median feature importance scores for the BRCA one-cancer models.These were scored using 100 repetitions of the model agnostic permutation feature importance algorithm [10]. The bars represent the distribution of scores between the lower and upper 5% quantiles of importance values from the repetitions.

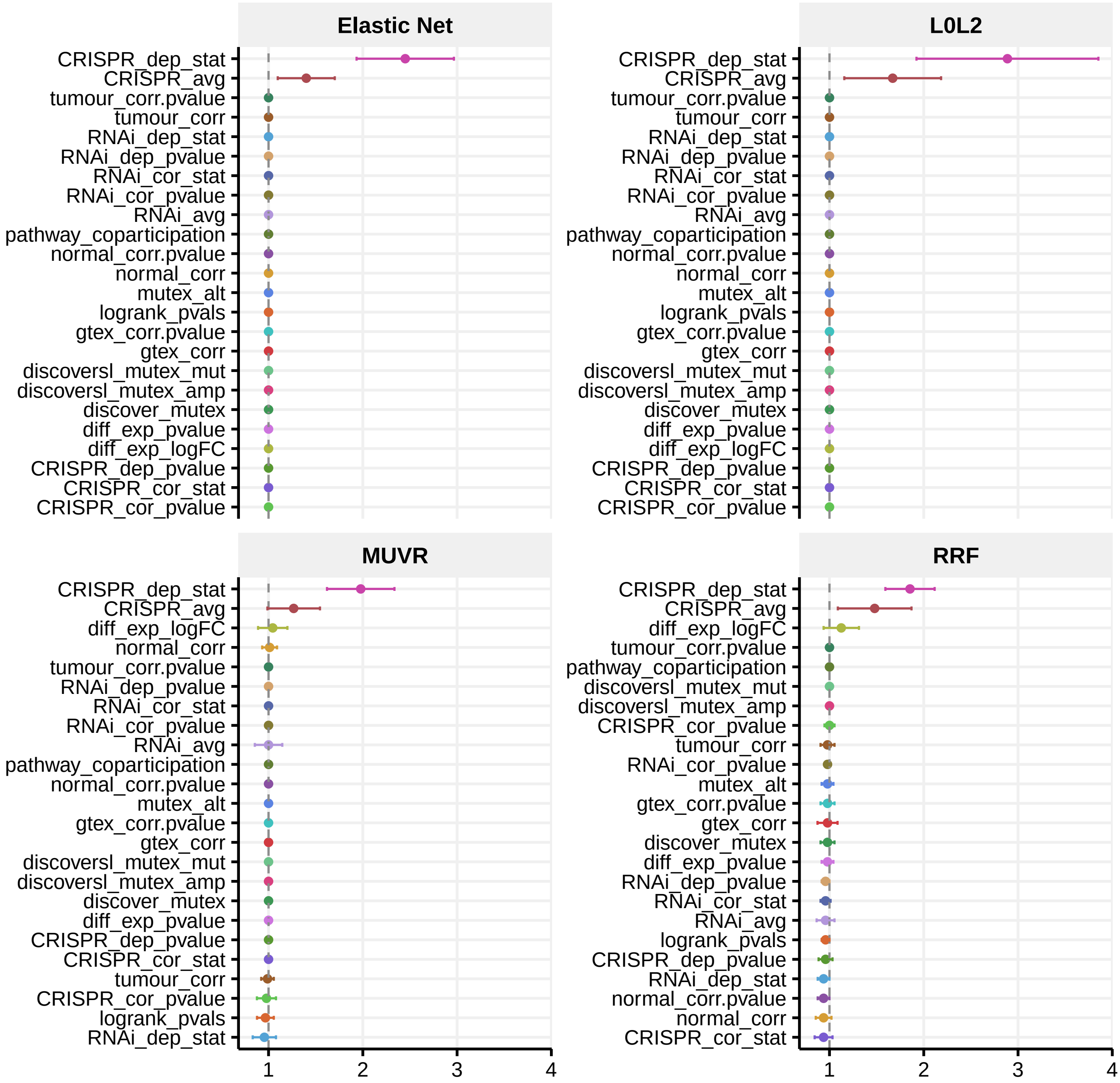

Figure S16: Median feature importance scores for the LUAD one-cancer models.These were scored using 100 repetitions of the model agnostic permutation feature importance algorithm [10]. The bars represent the distribution of scores between the lower and upper 5% quantiles of importance values from the repetitions.

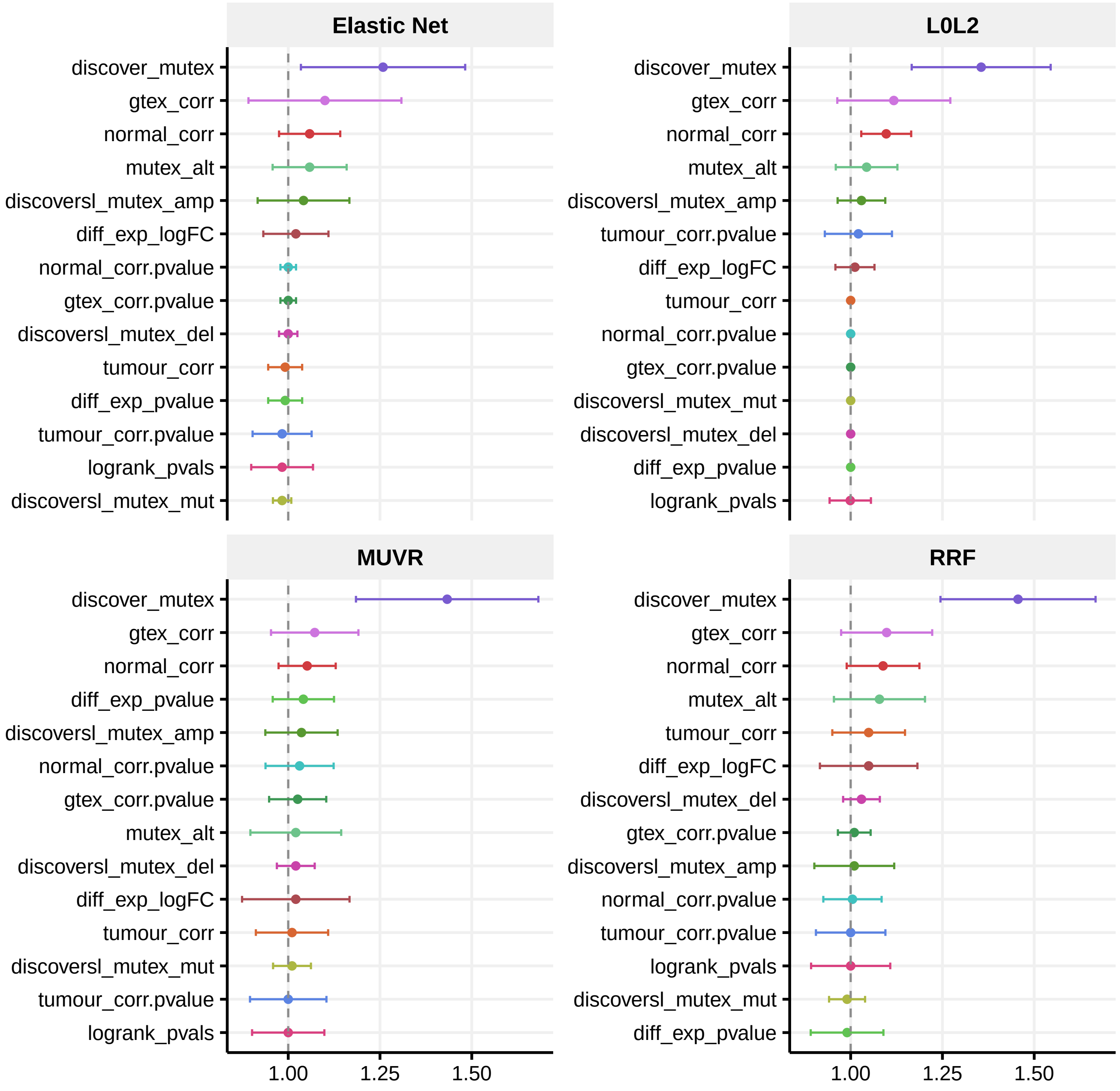

Figure S17: Median feature importance scores for the BRCA models trained without gene dependency-based features. These were scored using 100 repetitions of the model agnostic permutation feature importance algorithm [10]. The bars represent the distribution of scores between the lower and upper 5% quantiles of importance values from the repetitions..

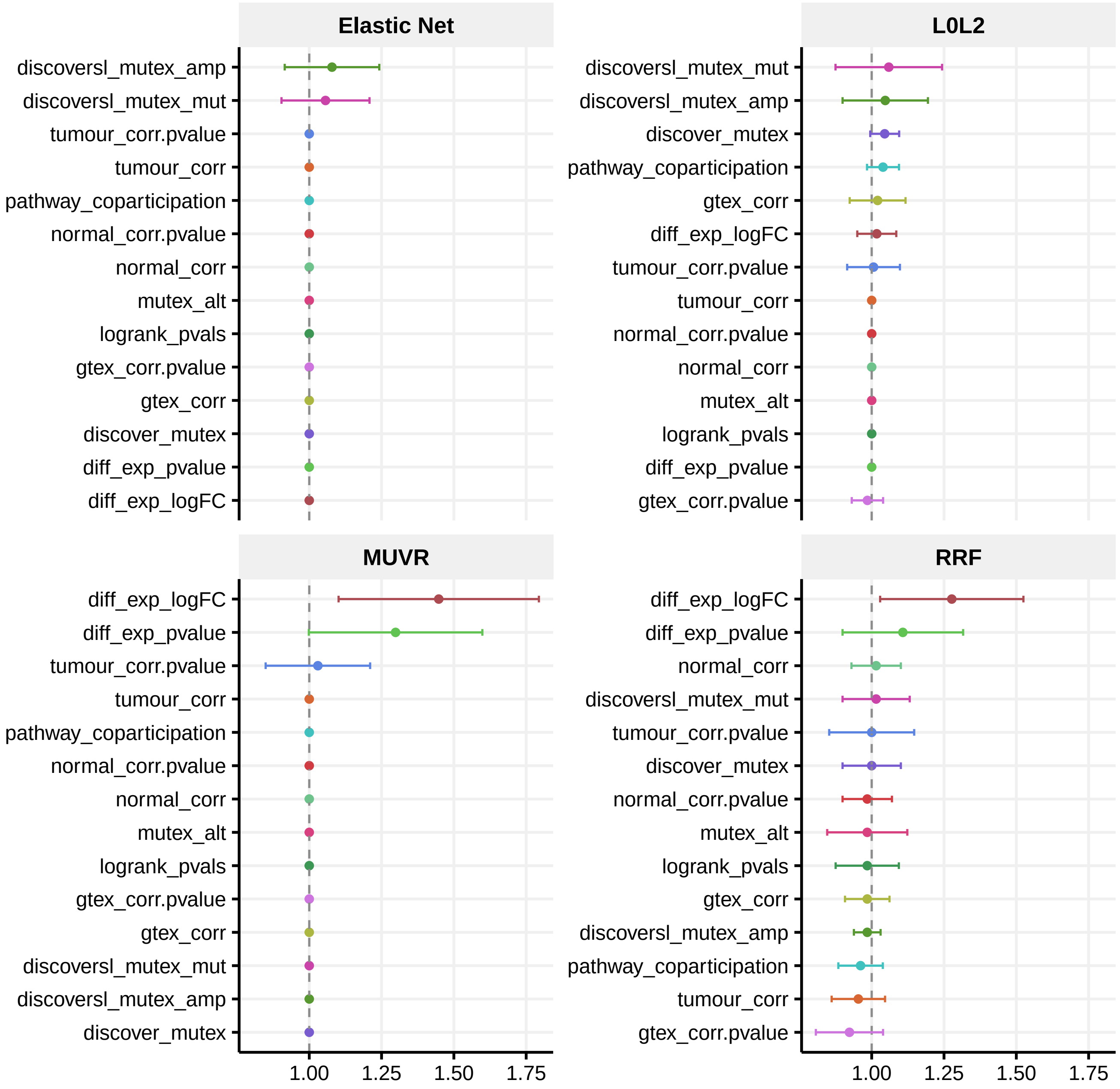

Figure S18: Median feature importance scores for the LUAD models trained without gene dependency-based features. These were scored using 100 repetitions of the model agnostic permutation feature importance algorithm [10]. The bars represent the distribution of scores between the lower and upper 5% quantiles of importance values from the repetitions.

References

[1] M. Ashburner, C. A. Ball, J. A. Blake, D. Botstein, H. Butler, J. M. Cherry, A. P. Davis, K. Dolinski, S. S. Dwight, J. T. Eppig, et al. Gene ontology: tool for the unification of biology. *Nature genetics*, 25(1):25–29, 2000.

[2] Ö. Babur, M. Gönen, B. A. Aksoy, N. Schultz, G. Ciriello, C. Sander, and E. Demir. Systematic identification of cancer driving signaling pathways based on mutual exclusivity of genomic alterations. *Genome Biology*, 16(1):45, 2015.

[3] V. Bewick, L. Cheek, and J. Ball. Statistics review 12: survival analysis. *Critical care (London, England)*, 8(5):389–394, 2004.

[4] S. Canisius, J. W. Martens, and L. F. Wessels. A novel independence test for somatic alterations in cancer shows that biology drives mutual exclusivity but chance explains most co-occurrence. *Genome Biology*, 2016.

[5] K. R. Cron, K. Zhu, D. S. Kushwaha, G. Hsieh, D. Merzon, J. Rameseder, C. C. Chen, A. D. D'Andrea, and D. Kozono. Proteasome inhibitors block dna repair and radiosensitize non-small cell lung cancer. *PloS one*, 8(9):e73710, 2013.

[6] S. Das, X. Deng, K. Camphausen, and U. Shankavaram. DiscoverSL: an R package for multi-omic data driven prediction of synthetic lethality in cancers. *Bioinformatics*, 35(4):701–702, 2019.

[7] J. M. Dempster, J. Rossen, M. Kazachkova, J. Pan, G. Kugener, D. E. Root, and A. Tsherniak. Extracting biological insights from the project achilles genome-scale crispr screens in cancer cell lines. *bioRxiv*, 2019. https://doi.org/10.1101/720243.

[8] H. Deng and G. Runger. Feature selection via regularized trees. In *The 2012 International Joint Conference on Neural Networks (IJCNN)*, pages 1–8. IEEE, 2012.

[9] T. Fawcett. An introduction to roc analysis. *Pattern Recognition Letters*, 27(8):861 – 874, 2006. ROC Analysis in Pattern Recognition.

[10] A. Fisher, C. Rudin, and F. Dominici. All models are wrong, but many are useful: Learning a variable's importance by studying an entire class of prediction models simultaneously. *Journal of Machine Learning Research*, 20(177):1–81, 2019.

[11] J. Friedman, T. Hastie, and R. Tibshirani. Regularization paths for generalized linear models via coordinate descent. *Journal of Statistical Software*, 33(1):1–22, 2010.

[12] M. Ghandi, F. W. Huang, J. Jané-Valbuena, G. V. Kryukov, C. C. Lo, E. R. McDonald, J. Barretina, E. T. Gelfand, C. M. Bielski, H. Li, K. Hu, A. Y. Andreev-Drakhlin, J. Kim, J. M. Hess, B. J. Haas, F. Aguet, B. A. Weir, M. V. Rothberg, B. R. Paolella, M. S. Lawrence, R. Akbani, Y. Lu, H. L. Tiv, P. C. Gokhale, A. de Weck, A. A. Mansour, C. Oh, J. Shih, K. Hadi, Y. Rosen, J. Bistline, K. Venkatesan, A. Reddy, D. Sonkin, M. Liu, J. Lehar, J. M. Korn, D. A. Porter, M. D. Jones, J. Golji, G. Caponigro, J. E. Taylor, C. M. Dunning, A. L. Creech, A. C. Warren, J. M. McFarland, M. Zamanighomi, A. Kauffmann, N. Stransky, M. Imielinski, Y. E. Maruvka, A. D. Cherniack, A. Tsherniak, F. Vazquez, J. D. Jaffe, A. A. Lane, D. M. Weinstock, C. M. Johannessen, M. P. Morrissey, F. Stegmeier, R. Schlegel, W. C. Hahn, G. Getz, G. B. Mills, J. S. Boehm, T. R. Golub, L. A. Garraway, and W. R. Sellers. Next-generation characterization of the Cancer Cell Line Encyclopedia. *Nature*, 569(7757):503–508, 2019.

[13] H. Hazimeh and R. Mazumder. Fast best subset selection: Coordinate descent and local combinatorial optimization algorithms. *Operations Research*, 68(5):1517–1537, 2020.

[14] J. Huang, M. Wu, F. Lu, L. Ou-Yang, and Z. Zhu. Predicting synthetic lethal interactions in human cancers using graph regularized self-representative matrix factorization. *BMC Bioinformatics*, 20(S19):657, 2019.

[15] G. James, D. Witten, T. Hastie, and R. Tibshirani. *An introduction to statistical learning*, volume 112. Springer, 2013.

[16] L. Jerby-Arnon, N. Pfetzer, Y. Y. Waldman, L. McGarry, D. James, E. Shanks, B. Seashore-Ludlow, A. Weinstock, T. Geiger, P. A. Clemons, E. Gottlieb, and E. Ruppin. Predicting Cancer-Specific Vulnerability via Data-Driven Detection of Synthetic Lethality. *Cell*, 158(5):1199–1209, 2014.

[17] A. Klami, G. Bouchard, and A. Tripathi. Group-sparse embeddings in collective matrix factorization. In *Proceedings of International Conference on Learning Representations (ICLR) 2014*, Apr. 2014. International Conference on Learning Representations ; Conference date: 14-04-2014 Through 16-04-2014.

[18] A. Lachmann, H. Xu, J. Krishnan, S. I. Berger, A. R. Mazloom, and A. Ma'ayan. Chea: transcription factor regulation inferred from integrating genome-wide chip-x experiments. *Bioinformatics*, 26(19):2438–2444, 2010.

[19] M. S. Lawrence, P. Stojanov, P. Polak, G. V. Kryukov, K. Cibulskis, A. Sivachenko, S. L. Carter, C. Stewart, C. H. Mermel, S. A. Roberts, et al. Mutational heterogeneity in cancer and the search for new cancer-associated genes. *Nature*, 499(7457):214–218, 2013.

[20] J. S. Lee, A. Das, L. Jerby-Arnon, R. Arafeh, N. Auslander, M. Davidson, L. McGarry, D. James, A. Amzallag, S. G. Park, K. Cheng, W. Robinson, D. Atias, C. Stossel, E. Buzhor, G. Stein, J. J. Waterfall, P. S. Meltzer, T. Golan, S. Hannenhalli, E. Gottlieb, C. H. Benes, Y. Samuels, E. Shanks, and E. Ruppin. Harnessing synthetic lethality to predict the response to cancer treatment. *Nature Communications*, 9(1):2546, 2018.

[21] H. Liany, A. Jeyasekharan, and V. Rajan. Predicting synthetic lethal interactions using heterogeneous data sources. *Bioinformatics*, 36(7):2209–2216, 2020.

[22] A. Liberzon, A. Subramanian, R. Pinchback, H. Thorvaldsdóttir, P. Tamayo, and J. P. Mesirov. Molecular signatures database (MSigDB) 3.0. *Bioinformatics*, 27(12):1739–1740, 2011.

[23] J. Liu, D. Guan, M. Dong, J. Yang, H. Wei, Q. Liang, L. Song, L. Xu, J. Bai, C. Liu, et al. Ufmylation maintains tumour suppressor p53 stability by antagonizing its ubiquitination. *Nature cell biology*, 22(9):1056–1063, 2020.

[24] Y. Long, M. Wu, Y. Liu, J. Zheng, C. K. Kwoh, J. Luo, and X. Li. Graph contextualized attention network for predicting synthetic lethality in human cancers. *Bioinformatics*, 2021. btab110.

[25] J. Lonsdale, J. Thomas, M. Salvatore, R. Phillips, E. Lo, S. Shad, R. Hasz, G. Walters, F. Garcia, N. Young, et al. The genotype-tissue expression (gtex) project. *Nature Genetics*, 45(6):580, 2013.

[26] T. D. Martin, D. R. Cook, M. Y. Choi, M. Z. Li, K. M. Haigis, and S. J. Elledge. A role for mitochondrial translation in promotion of viability in k-ras mutant cells. *Cell reports*, 20(2):427–438, 2017.

[27] J. M. McFarland, Z. V. Ho, G. Kugener, J. M. Dempster, P. G. Montgomery, J. G. Bryan, J. M. Krill-Burger, T. M. Green, F. Vazquez, J. S. Boehm, T. R. Golub, W. C. Hahn, D. E. Root, and A. Tsherniak. Improved estimation of cancer dependencies from large-scale RNAi screens using model-based normalization and data integration. *Nature Communications*, 9(1), 2018.

[28] C. H. Mermel, S. E. Schumacher, B. Hill, M. L. Meyerson, R. Beroukhim, and G. Getz. Gistic2. 0 facilitates sensitive and confident localization of the targets of focal somatic copy-number alteration in human cancers. *Genome Biology*, 12(4):R41, 2011.

[29] R. M. Meyers, J. G. Bryan, J. M. McFarland, B. A. Weir, A. E. Sizemore, H. Xu, N. V. Dharia, P. G. Montgomery, G. S. Cowley, S. Pantel, A. Goodale, Y. Lee, L. D. Ali, G. Jiang, R. Lubonja, W. F. Harrington, M. Strickland, T. Wu, D. C. Hawes, V. A. Zhivich, M. R. Wyatt, Z. Kalani, J. J. Chang, M. Okamoto, K. Stegmaier, T. R. Golub, J. S. Boehm, F. Vazquez, D. E. Root, W. C. Hahn, and A. Tsherniak. Computational correction of copy number effect improves specificity of CRISPR–Cas9 essentiality screens in cancer cells. *Nature Genetics*, 49(12):1779–1784, 2017.

[30] D. G. Pestov, Z. Strezoska, and L. F. Lau. Evidence of p53-dependent cross-talk between ribosome biogenesis and the cell cycle: effects of nucleolar protein bop1 on g1/s transition. *Molecular and cellular biology*, 21(13):4246–4255, 2001.

[31] M. Rahman, L. K. Jackson, W. E. Johnson, D. Y. Li, A. H. Bild, and S. R. Piccolo. Alternative preprocessing of RNA-Sequencing data in The Cancer Genome Atlas leads to improved analysis results. *Bioinformatics*, 31(22):3666–3672, 2015.

[32] M. D. Robinson, D. J. McCarthy, and G. K. Smyth. edger: a bioconductor package for differential expression analysis of digital gene expression data. *Bioinformatics*, 26(1):139–140, 2010.

[33] L. Shi, J. A. Westerhuis, J. Rosén, R. Landberg, and C. Brunius. Variable selection and validation in multivariate modelling. *Bioinformatics*, 35(6):972–980, 2019.

[34] C. Stark, B.-J. Breitkreutz, T. Reguly, L. Boucher, A. Breitkreutz, and M. Tyers. Biogrid: a general repository for interaction datasets. *Nucleic acids research*, 34(suppl_1):D535–D539, 2006.

[35] D. Szklarczyk, A. L. Gable, K. C. Nastou, D. Lyon, R. Kirsch, S. Pyysalo, N. T. Doncheva, M. Legeay, T. Fang, P. Bork, et al. The string database in 2021: customizable protein–protein networks, and functional characterization of user-uploaded gene/measurement sets. *Nucleic acids research*, 49(D1):D605–D612, 2021.

[36] A. Tsherniak, F. Vazquez, P. G. Montgomery, B. A. Weir, G. Kryukov, G. S. Cowley, S. Gill, W. F. Harrington, S. Pantel, J. M. Krill-Burger, R. M. Meyers, L. Ali, A. Goodale, Y. Lee, G. Jiang, J. Hsiao, W. F. J. Gerath, S. Howell, E. Merkel, M. Ghandi, L. A. Garraway, D. E. Root, T. R. Golub, J. S. Boehm, and W. C. Hahn. Defining a Cancer Dependency Map. *Cell*, 170:564–576.E16, 2017.

[37] Y. Yoshida, T. Tsunoda, K. Doi, Y. Tanaka, T. Fujimoto, T. Machida, T. Ota, M. Koyanagi, Y. Takashima, T. Sasazuki, et al. Kras-mediated up-regulation of rrm2 expression is essential for the proliferation of colorectal cancer cell lines. *Anticancer research*, 31(7):2535–2539, 2011.
